# Chamigrane Sesquiterpenes from a Basidiomycetous Endophytic Fungus XG8D Associated with Thai Mangrove *Xylocarpus granatum*

**DOI:** 10.3390/md14070132

**Published:** 2016-07-15

**Authors:** Siwattra Choodej, Thapong Teerawatananond, Tohru Mitsunaga, Khanitha Pudhom

**Affiliations:** 1 The United Graduate School of Agricultural Science, Gifu University, 1-1 Yanagido, Gifu 501-1193, Japan; csiwattra@hotmail.com (S.C.); mitunaga@gifu-u.ac.jp (T.M.); 2 Faculty of Science and Technology, Valaya Alongkorn Rajabhat University under Royal Patronage, Pathumtani 13138, Thailand; thapong_sthc@hotmail.com; 3 Department of Chemistry, Faculty of Science, Chulalongkorn University, Bangkok 10330, Thailand

**Keywords:** chamigrane, sesquiterpene, endophytic fungus, cytotoxic

## Abstract

Six new chamigrane sesquiterpenes, merulinols A‒F (**1**‒**6**), and four known metabolites (**7**‒**10**) were isolated from the culture of the basidiomycetous fungus XG8D, a mangrove-derived endophyte. Their structures were elucidated mainly by 1D and 2D NMR, while the structures of **1** and **2** were further confirmed by single-crystal X-ray diffraction analysis. The in vitro cytotoxicity of all compounds was evaluated against three human cancer cell lines, MCF-7, Hep-G2, and KATO-3. Compounds **3** and **4** selectively displayed cytotoxicity against KATO-3 cells with IC_50_ values of 35.0 and 25.3 μM, respectively.

## 1. Introduction

Marine-associated microorganisms are recognized as a promising source of chemically diverse, structurally unique, and biologically active secondary metabolites [1,2]. Among them, the fungi derived from mangrove plants are of special interest and continue to attract considerable attention due to the extreme environmental conditions [3,4,5,6,7,8]. Our research group has, therefore, focused on the exploration of bioactive natural products from these types of fungi collected from mangrove areas in Thailand [9,10]. The isolation and characterization of unique sesquiterpene endoperoxides of the chamigrane type was previously reported from the ethyl acetate (EtOAc) extract of the marine-derived fungal strain XG8D, grown in corn-steep liquor-containing medium [11,12]. This fungus was obtained from the leaves of the mangrove plant *Xylocarpus granatum*. Chamigrane sesquiterpenoids, mostly halogenated, are generally produced by the red alga in the genus *Laurencia* (Family Rhodomelaceae) [13,14,15,16,17], with those from fungi being rare [18]. The only precedents include merulins A–D from the basidiomycetous endophytic fungus XG8D [11,12], steperoxide A from the higher fungus *Steccherinum ochraceum* [19], talaperoxides A–D from the mangrove endophytic fungus *Talaromyces flavus* [20], and acaciicolinolides A–C and acaciicolinols A–L from *Pseudolagarobasidium acaciicola*, which is also a mangrove-derived endophytic fungus [21].

**Figure 1:**
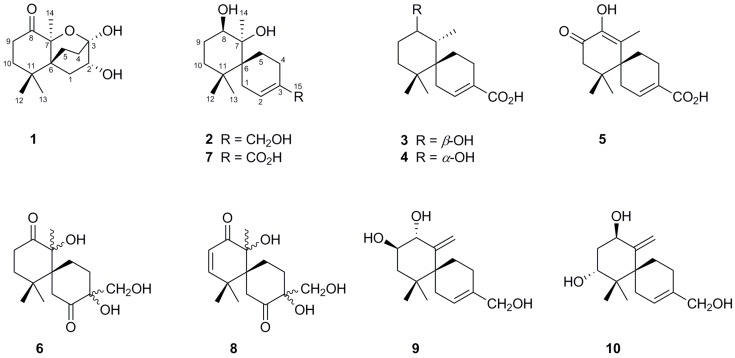
Structures of metabolites **1**–**10** isolated from the basidiomycetous fungus XG8D.

Moreover, some studies have shown that the production of secondary metabolites might be highly dependent on fermentation conditions and modes [22,23,24]. In particular, endophytic fungi seem to be affected to a greater extent by variations in the culture media, which leads to changes in their metabolite profiles [25]. This prompted us to embark on a study of the effect of the culture medium on the metabolite production of the endophytic fungus strain XG8D. As a result, the primary screening of the cultivation conditions for this strain indicated that alteration of the culture medium greatly affected the chemical profile of this fungus. In the present study, the chemical investigation of the XG8D strain grown in Sabouraud dextrose broth (SDB) led to the isolation of six new chamigrane sesquiterpenes, not of the endoperoxide type, namely merulinols A–G (**1**–**6**), and four known compounds, acaciicolinols C (**7**), K (**8**), F (**9**) and D (**10**) (Figure 1). Details of the isolation, structure elucidation and in vitro cytotoxic activity of these metabolites are reported herein.

## 2. Results and Discussion

**Figure 2:**
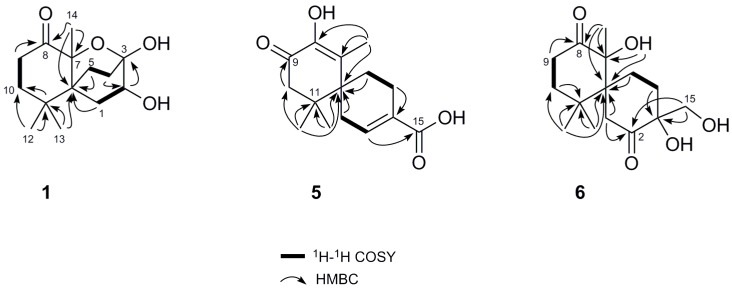
^1^H‒^1^H COSY and selected HMBC correlations of **1**, **5**, and **6**.

**Figure 3:**
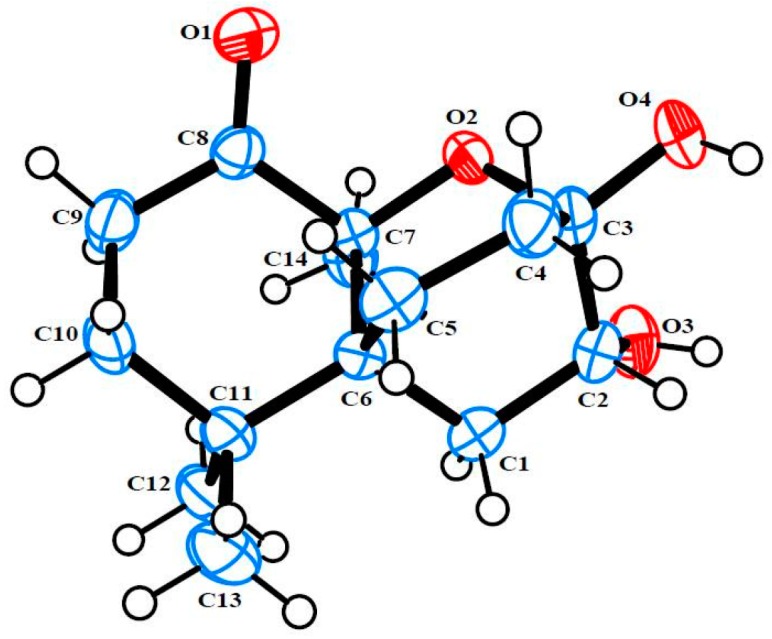
ORTEP plot of **1**.

Merulinol A (**1**) was obtained as colorless crystals and was determined to have a molecular formula of C_14_H_22_O_4_ by the HR-ESIMS ion at *m/z* 277.1419 [M + Na]^+^, implying four degrees of unsaturation. Combined analysis of the ^1^H and ^13^C NMR (Table 1, Figures S1–S3) plus the HSQC (Figure S4) spectroscopic data revealed the presence of three singlet methyls including one downfield signal at δ_H_ 1.68, five *sp*^3^ methylenes, one oxymethine and five quaternary carbons, including one ketone carbon (δ_C_ 212.0). These data accounted for all the NMR resonances of **1** and one of three unsaturations, indicating that **1** was a tricyclic compound. Analysis of the ^1^H–^1^H COSY spectrum (Figure 2 and Figure S3) established three partial structures of H_2_-1/H-2, H_2_-4/ H_2_-5, and H_2_-9/H_2_-10. The C-10 methylene and C-6 quaternary carbon were connected to C-11 by the HMBC correlations from H_2_-10, H_3_-12, and H_3_-13 to C-11, as well as from H_3_-12 and H_3_-13 to C-6 (Figure 2 and Figure S5). Further HMBC cross-peaks from a downfield singlet methyl at δ_H_ 1.68 to the C-8 ketone carbon, C-7 oxygenated quaternary carbon and C-6 led to the attachment of this methyl group (C-14) on C-7 and the connection of C-6 and C-8 on C-7. Two other partial structures, H_2_-1/H-2 and H_2_-4/ H_2_-5, were placed between C-6 and C-3 from the HMBC correlations of H_2_-1, H-2, H_2_-4, H_2_-5 to C-6 and from H-2 to C-3, and so established a 6,6-bicyclic skeleton via a C-6 spiro carbon. Moreover, the ^13^C resonance of C-3 at δ_C_ 94.5 implied that C-3 was a hemiketal moiety. According to the molecular formula C_14_H_22_O_4_, and the degree of unsaturation of **1**, C-3 was thus linked, through an ether bond, to C-7 to establish a tricyclic structure as shown. The proposed structure of **1** and its relative configuration were further confirmed by single-crystal X-ray diffraction analysis using Mo Kα radiation, and a perspective ORTEP plot is depicted in Figure 3.

**Figure 4:**
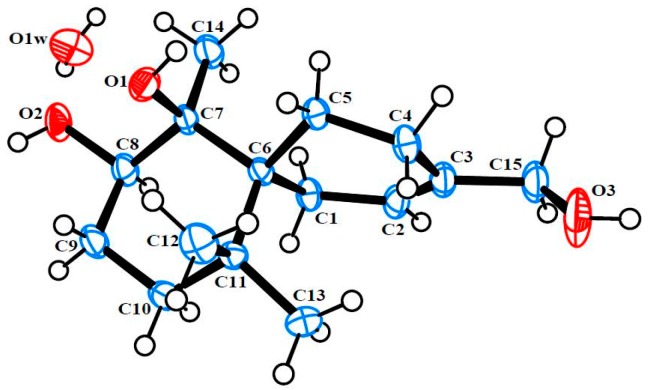
ORTEP plot of **2**.

Merulinol B (**2**) was also obtained as colorless crystals. HR-ESIMS analysis of the molecular ion (*m/z* 277.1771 [M + Na]^+^) suggested the molecular formula of C_15_H_26_O_3_, which was indicative of three degrees of unsaturation. Analysis of ^1^H and ^13^C NMR (Table 1, Figures S6–S8) plus the HSQC (Figure S9) spectroscopic data indicated the presence of three singlet methyls, six *sp*^3^ methylenes (one oxymethylene), one oxymethine, one olefinic methine and four quaternary carbons (one oxygenated and one olefinic carbon). It was revealed that compound **2** had similar ^1^H and ^13^C NMR spectra to those of acaciicolinol C (**7**) [21], except the C-15 carboxylic group in **7** was replaced by a hydroxymethyl in **2**. Moreover, an exchangeable proton at δ_H_ 3.08 (s) was assigned to 7-OH by its correlations to C-6 and C-7 (Figure S10). Finally, the relative configuration of **2** was assigned by NOESY correlation (Figure S11) and single-crystal X-ray diffraction analysis using Mo Kα radiation, and a perspective ORTEP plot is depicted in Figure 4.

**Figure 5:**
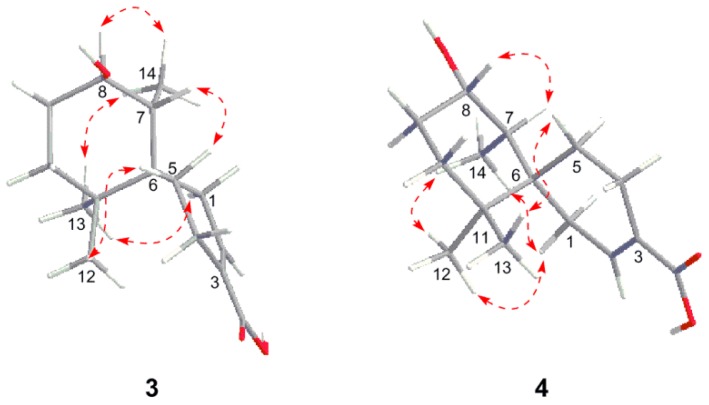
Selected NOESY correlations of **3** and **4**.

Merulinol C (**3**), obtained as a pale yellow gum, was assigned a molecular formula of C_15_H_24_O_3_ by HR-ESIMS (*m/z* 275.1612 [M + Na]^+^), implying four degrees of unsaturation. The ^1^H and ^13^C NMR spectroscopic data of **3** (Table 1, Figures S12–S15) closely resembled those of **2**. The obvious difference in the ^1^H NMR spectra between **2** and **3** was the presence of a doublet methyl signal at δ_H_ 1.01 (*J* = 6.8 Hz) in **3**, replacing the singlet methyl signal (C-14) in **2**. In addition, the ^13^C NMR signal of an oxyquaternary carbon (δ_C_ 78.3, C-7) in **2** was absent in **3**, while signals for a methine group (δ_H_ 1.65 m, δ_C_ 44.9) were observed in **3**. The location of the doublet methyl at C-14 was confirmed by its ^1^H–^1^H COSY correlation with H-7 (Figure S14) and HMBC cross-peaks from H_3_-14 to C-8, C-7 and C-6 (Figure S16). The NOESY correlations of H_3_-13/H_3_-14, H_3_-13/H_2_-1 and H_3_-14/H_2_-1 (Figure 5 and Figure S17) supported that **3** had the same relative configuration as **2**. Additionally, the NOE correlation between H_3_-14 and H-8, as well as the lack of a correlation between H-7 and H-8, revealed the β-orientation of 8-OH.

Merulinol D (**4**) was obtained as a pale yellow gum. Its molecular formula was established as C_15_H_24_O_3_ by HR-ESIMS (*m/z* 275.1616 [M + Na]^+^), being the same as that of **3**. Its ^1^H and ^13^C NMR spectra (Table 2, Figures S18 and S19) displayed resonances that were nearly identical with those of **3**. Interpretation of 2D NMR data (Figures S20–S23) established the same planar structure as **3**. The NOESY correlations of **4** indicated that **4** had the same configuration as **3**, except for that of C-8. The observed NOE correlation between H-7 and H-8 (Figure 5) suggested the α-orientation of HO-8. Therefore, compound **4** was assigned as the C-8 epimer of **3**. This assignment was supported by the opposite signs of their specific rotations (${\left[\alpha \right]}_{D}^{20}$ +6.4 for **3** vs. −47.2 for **4**).

Merulinol E (**5**) was obtained as a yellow gum. The molecular formula was determined to be C_15_H_20_O_4_ by the HR-ESIMS ion at *m/z* 265.1431 [M + H]^+^, implying six degrees of unsaturation. The ^1^H and ^13^C NMR (Table 2, Figures S24–S26) plus the HSQC (Figure S27) data indicated the structure of **5** contained three singlet methyls, four methylenes, one olefinic methine, five quaternary carbons (including two olefinic) and two carbonyls. The two carbonyl signals at δ_C_ 192.3 and 170.9 were ascribed to one α,β-unsaturated ketone and one carboxylic acid carbonyl, respectively. Comparison of the NMR data of **5** with those of **3** and **4** revealed that **5** had the same B ring with an α,β-unsaturated carboxylic acid moiety as found in both **3** and **4**. Analysis of the HMBC spectrum indicated that an α,β-unsaturated ketone was located at C-7 to C-9 due to the correlations of H_3_-14/C-7, H_3_-14/C-8 and H_2_-10/C-9 (Figure 2 and Figure S28). The C-8 resonance (δ_C_ 135.9) was deshielded, which coupled with the HRESIMS data led to the attachment of the hydroxy group on this olefinic carbon. Indeed, the NMR data of **5** were similar to those of acaciicolinol L [21], except the C-15 hydroxymethyl in acaciicolinol L was replaced by a carboxylic moiety in **5**. The NOESY data (Figure S29) verified that **5** shared the same configuration as merulinols A‒E. Consequently, the structure of **5** was established as shown.

Merulinol F (**6**), also obtained as a pale yellow gum, had a molecular formula of C_15_H_24_O_5_ from the HR-ESIMS ion at *m/z* 307.1516 [M + Na]^+^. The ^1^H NMR spectrum (Figure S30) showed signals of three singlet methyls, five methylenes and one hydroxymethyl. The ^13^C NMR spectrum (Figure S31) exhibited two ketone resonances at δ_C_ 213.8 and 215.8. The NMR data of **6** (Figures S30–S34) were similar to those of acaciicolinol K (**9**) [21], except that the C-9–C-10 double bond in **9** was replaced by a single bond. This was confirmed by the ^1^H‒^1^H COSY correlation of H_2_-9 and H_2_-10 and the HMBC correlations from H_2_-9 to a ketone at δ_C_ 213.8 (C-8) (Figure 2 and Figures S32 and S34). Based on these spectroscopic data, the structure of **6** was determined as shown. However, its configuration at C-3 and C-7 could not be conclusively established from the NOESY data.

The in vitro cytotoxic activities of the isolated compounds **1**‒**10** against the MCF-7, Hep-G2, and KATO-3 cell lines were tested using MTT assay to approximate the number of viable cells, while doxorubicin was used as a positive control. Compounds **3** and **4** exhibited activities against KATO-3 cells with IC_50_ values of 35.0 ± 1.20 and 25.3 ± 0.82 μM, respectively, but against the other two cell lines and all the other compounds, a growth inhibition against the three cell lines of less than 50% at a dose of 50 μM was observed and thus they were considered inactive.

## 3. Experimental Section

### 3.1. General Experimental Procedures

Melting points were determined on a melting point M565 apparatus and are uncorrected. Optical rotations were measured on a JASCO P-1010 polarimeter (JASCO Corporation, Tokyo, Japan). UV spectra were recorded on a Spekol 1200 Analytic Jena spectrophotometer. IR spectra were recorded on a Bruker ALPHA FT-IR spectrometer (Bruker, MA, USA). NMR spectra were acquired on a Varian Mercury-400 Plus NMR spectrometer (Varian, CA, USA). High-resolution ESI mass spectroscopy was measured on a Bruker micrOTOF (Bruker). Silica gel (230–400 mesh, Merck, Darmstadt, Germany) and Sephadex LH20 (Amersham Biosciences, NJ, USA) were used for open-column chromatography. Analytical thin layer chromatography (TLC) was performed using precoated silica gel 60 GF254 plates (Merck). Single-crystal X-ray diffraction analysis was performed on a Bruker APEX II diffractometer (Bruker).

### 3.2. Fungal Material

The endophytic fungus XG8D used in this study was isolated from the healthy leaves of *Xylocarpus granatum* collected from Samutsakorn province, Thailand, in July 2008. The fungus was identified to the family Meruliaceae (order Polyporales, subclass Incertaesedis, class Agaricomycetes, phylum Basidiomycota) by analysis of the 28S rDNA and ITS data (GenBank accession Nos. HM060640 and HM060641).

### 3.3. Fermentation, Extraction, and Isolation

The fungus XG8D was cultured on potato dextrose agar at room temperature for 10 days. Then, five pieces (0.5 cm^2^ × 0.5 cm^2^) of agar plugs were inoculated in a 1000 mL Erlenmeyer flask containing 200 mL of Sabouraud dextrose broth (SDB) at 30 °C for 21 days. The fungal culture (10 L) was filtered to remove mycelia, and the culture broth was extracted twice with ethyl acetate (EtOAc). The combined EtOAc extract was concentrated under reduced pressure to afford a dark brown residue (12.60 g).

The EtOAc extract was subjected to column chromatography (CC) over Sephadex LH20 and eluted with MeOH to obtain five fractions (A–E). Fraction E (4.68 g) was fractionated by CC over silica gel eluting with a 1:2 to 1:0 gradient of EtOAc-*n*-hexane to give 11 fractions (E1–E11). Fraction E4 (379.4 mg) was separated by CC using MeOH-CH_2_Cl_2_ (1:19) to afford nine subfractions (E4.1–E4.9). Subfraction E4.8 was further purified using the same procedure as fraction E4 to provide **3** (5.2 mg), while subfraction E4.9 was eluted with acetone-CH_2_Cl_2_ (1:2) on silica gel column to afford **4** (2.8 mg). Fraction E5 (256.0 mg) was subjected to CC over silica gel using EtOAc-CH_2_Cl_2_ (3:2) to give seven subfractions (E5.1–E5.7). Subfraction E5.3 was purified by CC over Sephadex LH20 eluted with MeOH to provide **5** (3.7 mg). Fraction E8 (198.2 mg) was chromatographed on silica gel (acetone-*n*-hexane, 1:3) to afford six subfractions (E8.1–E8.6). Subfraction E8.4 gave **6** (5.2 mg) and **8** (25.2 mg) upon CC over silica gel (acetone-CH_2_Cl_2_, 2:8). Subfraction E8.6 (52.6 mg) was further purified by a silica gel CC using EtOAc-CH_2_Cl_2_ (1:1) to yield a pale yellow solid, followed by recrystallization with MeOH to give **7** (16.2 mg). Subfraction E8.5 (75.3 mg) was subjected to CC over silica gel using MeOH-CH_2_Cl_2_ (1:19) to obtain five fractions (E8.5.1–E8.5.5). After solvent evaporation, a light yellow solid was formed from fraction E8.5.5, which was further recrystallized with MeOH to afford **2** (9.8 mg). Fraction E9 was purified by silica gel CC using 2:3 EtOAc-benzene elution followed by recrystallization from MeOH to yield **1** (2.4 mg). Fraction E10 (239.4 mg) was subjected to CC over silica gel using MeOH-CH_2_Cl_2_ (1:19, then 1:9) to provide 14 subfractions (E10.1–E10.14). Subfractions E10.11 (12.8 mg) and E10.14 (10.1 mg) were further purified by Sephadex LH20 CC eluting with MeOH to afford **9** (5.6 mg) and **10** (3.3 mg), respectively.

*Merulinol A* (**1**): colorless crystals; mp 195–199 °C, ${\left[\alpha \right]}_{D}^{20}$ +18.4 (*c* 0.1, MeOH); UV (MeOH) λ_max_ (log ε) 218 (2.71), 279 (2.31) nm; IR (neat) ν_max_ 3413, 2969, 1718, 1460, 1375, 1217, 1049 cm^–1^; ^1^H and ^13^C NMR data (Table 1); HR-ESIMS *m/z* 277.1419 [M + Na]^+^ (calcd. for C_14_H_22_O_4_Na, *m/z* 277.1416 [M + Na]^+^).

*Merulinol B* (**2**): colorless crystals; mp 126–129 °C, ${\left[\alpha \right]}_{D}^{20}$ ‒16.8 (*c* 0.1, MeOH); UV (MeOH) λ_max_ (log ε) 213 (2.25), 239 (2.25) nm; IR (neat) ν_max_ 3359, 2924, 2853, 1738, 1658, 1632, 1468, 1367, 1216, 1054 cm^–1^; ^1^H and ^13^C NMR data (Table 1); HR-ESIMS *m/z* 277.1771 [M + Na]^+^ (calcd. for C_15_H_26_O_3_Na, *m/z* 277.1780 [M + Na]^+^).

*Merulinol C* (**3**): Pale yellow gum; ${\left[\alpha \right]}_{D}^{20}$ +5.6 (*c* 0.1, MeOH); UV (MeOH) λ_max_ (log ε) 225 (3.06) nm; IR (neat) ν_max_ 3441, 2925, 2855, 1737, 1685, 1457, 1367, 1217, 1024 cm^–1^; ^1^H and ^13^C NMR data (Table 1); HR-ESIMS *m/z* 275.1612 [M + Na]^+^ (calcd. for C_15_H_24_O_3_Na, *m/z* 275.1623 [M + Na]^+^).

*Merulinol D* (**4**): Pale yellow gum; ${\left[\alpha \right]}_{D}^{20}$ ‒47.2 (*c* 0.1, MeOH); UV (MeOH) λ_max_ (log ε) 227 (2.98) nm; IR (neat) ν_max_ 3435, 2925, 2856, 1732, 1687, 1455, 1360, 1223, 1021 cm^–1^; ^1^H and ^13^C NMR data (Table 2); HR-ESIMS *m/z* 275.1616 [M + Na]^+^ (calcd. for C_15_H_24_O_3_Na, *m/z* 275.1623 [M + Na]^+^).

*Merulinol E* (**5**): Pale yellow gum; ${\left[\alpha \right]}_{D}^{20}$ +127.2 (*c* 0.1, MeOH); UV (MeOH) λ_max_ (log ε) 218 (2.82), 280 (2.82) nm; IR (neat) ν_max_ 3361, 2923, 2852, 1738, 1658, 1468, 1366, 1217 cm^–1^; ^1^H and ^13^C NMR data (Table 2); HR-ESIMS *m/z* 265.1431 [M + H]^+^ (calcd. for C_15_H_21_O_4_, *m/z* 265.1440 [M + H]^+^).

*Merulinol F* (**6**): Pale yellow gum; ${\left[\alpha \right]}_{D}^{20}$ ‒138.0 (*c* 0.1, MeOH); UV (MeOH) λ_max_ (log ε) 215 (2.49), 291 (2.05) nm; IR (neat) ν_max_ 3388, 2927, 2855, 1706, 1659, 1367, 1230 cm^–1^; ^1^H and ^13^C NMR data (Table 2); HR-ESIMS *m/z* 307.1516 [M + Na]^+^ (calcd. for C_15_H_24_O_5_Na, *m/z* 307.1521 [M + Na]^+^).

### 3.4. Single X-ray Crystallograpic Analysis of Merulinols A (**1**) and B (**2**)

All crystallographic data were collected at 293 K on a Bruker APEX II diffractometer with Mo Kα radiation (λ = 0.71073). The structures were solved by direct methods using SHELXS-97 and refined by full-matrix least-squares on all *F*^2^ data using SHELXS-97 to final *R* values [26,27]. All hydrogen atoms were added at calculated positions and refined using a rigid model. Crystallographic data for **1** and **2** have been deposited with the Cambridge Crystallographic Data Centre with the deposition numbers CCDC 1404677 and 832629, respectively [28].

*Crystal Data for **1**:* colorless crystal, C_14_H_22_O_4_, *M*_r_ = 254.32, orthorhombic, *a* = 9.0809(17) Å, *b* = 10.223(2) Å, *c* = 13.933(4) Å, space group *P*2_1_2_1_2_1_, *Z* = 4, *D*_x_ = 1.306 Mg/m^3^, and *V* = 1293.5(5) Å^3^, μ (Mo Kα) = 0.09 mm^‒1^, and *F*(000) = 552. Crystal dimensions: 0.40 mm × 0.25 mm × 0.20 mm. Independent reflections: 1901 (*R*_int_ = 0.022). The final *R*_1_ values were 0.042, *wR*_2_ = 0.103 (*I* > 2σ(*I*)).

*Crystal Data for **2**:* colorless crystal, C_30_H_48_O_8_, *M*_r_ = 536.68, orthorhombic, *a* = 11.3273(7) Å, *b* = 11.8750(7) Å, *c* = 20.5308(13) Å, space group *P*2_1_2_1_2_1_, *Z* = 4, *D_x_* = 1.291 Mg/m^3^, and *V* = 2761.6(3) Å^3^, μ (Mo Kα) = 0.09 mm^‒1^, and *F*(000) = 1168. Crystal dimensions: 0.40 mm× 0.30 mm× 0.18 mm. Independent reflections: 5564 (*R*_int_ = 0.112). The final *R*_1_ values were 0.095, *wR*_2_ = 0.268 (*I* > 2σ(*I*)).

### 3.5. Cell Culture

Human breast (MCF-7), liver (Hep-G2), and gastric (KATO-3) cancer cell lines were cultured in RPMI-1640 (HiMedia Laboratories, Mumbai, India) supplemented with 100 U/mL of penicillin, 100 μg/mL of streptomycin and 10% fetal bovine serum (FBS, Thermo Scientific, Waltham, MA, USA). The cells were incubated in a humidified atmosphere of 5% CO_2_ at 37 °C and sub-cultured every three days.

### 3.6. Cytotoxicity Assay

Cytotoxicity of isolated compounds against the MCF-7, Hep-G2 and KATO-3 cancer cells was assessed using the MTT (3-[4,5-dimethylthiazol-2-yl-2,5-diphenyltetrazolium] bromide) assay as previously described [29]. Briefly, freshly trypsinized cell suspensions were seeded into 96-well culture plates at 1 × 10^4^ cells/well in the presence or absence of the test compound. After incubation at 37 °C for 72 h, 10 μL of MTT solution (5 mg in PBS 1 mL) was added to each well for 4 h. The cell-free supernatant was then removed, and DMSO was added to dissolve the formazan crystals. Absorbance values were measured with a microplate reader at 540 nm. Experiments were operated in triplicate, and data are described as mean ± SD of three independent experiments. Doxorubicin was used as a positive control.

## 4. Conclusions

As a result, six new fungal metabolites in the class of chamigrane sesquiterpenes (**1**‒**6**), together with four known compounds (**7**‒**10**), were isolated from the basidiomycetous endophytic fungus strain XG8D which was isolated from a Thai mangrove plant. Among the isolated compounds, merulinol A (**1**) is a nor-chamigrane with a novel tricyclic ring system, whereas compounds **2**‒**10** are 6/6 spirobicyclic charmigrane sesquiterpenes. Compounds **3** and **4** selectively exhibited cytotoxicity toward gastric KATO-3 cells with IC_50_ values of 35.0 ± 1.20 and 25.3 ± 0.82 μM, respectively.

## Figures and Tables

**Table 1:** ^1^H (400 MHz) and ^13^C NMR (100 MHz) data of compounds **1**‒**3**.

Position	1 ^a^		2 ^b^		3 ^a^	
	δ_C_, Type	δ_H_, Mult. (*J* in Hz)	δ_C_, Type	δ_H_, Mult. (*J* in Hz)	δ_C_, Type	δ_H_, Mult. (*J* in Hz)
1	32.4, CH_2_	1.99, m	29.2, CH_2_	2.19, d (20.0)	26.9, CH_2_	2.15, m
		2.11, dt (14.4, 2.8)		2.34, d (20.0)		
2	69.4, CH	3.85, dd (9.6, 2.0)	124.0, CH	5.16, br s	144.1, CH	7.18, br s
3	94.5, qC		137.7, qC		130.0, qC	
4	29.5, CH_2_	1.66, m	25.1, CH_2_	1.89, m	23.3, CH_2_	2.24, m
		1.94, m				2.44, m
5	25.7, CH_2_	1.64, m	25.8, CH_2_	2.05, m	29.1, CH_2_	1.63, m
		1.70, m				
6	43.5, qC		44.3, qC		41.3, qC	
7	86.0, qC		78.3, qC		44.9, CH	1.65, m
8	212.0, qC		76.9, CH	3.56, br s	72.5, CH	3.33, td (12.0, 4.0)
9	35.1, CH_2_	2.31, m	26.9, CH_2_	1.38, dq (12.0, 4.0)	31.6, CH_2_	1.48,m
		2.75, m		2.10, m		1.79, dq (12.0, 4.0)
10	37.3, CH_2_	1.61, m	34.7, CH_2_	1.00, m	36.6, CH_2_	1.23, dt (12.0, 4.0)
		1.92, m		1.86, m		1.60, m
11	37.1, qC		38.3, qC		37.3, qC	
12	27.3, CH_3_	0.94, s	30.5, CH_3_	0.85, s	27.9, CH_3_	0.78, s
13	25.1, CH	1.24, s	26.3, CH_3_	1.17, s	22.5, CH_3_	0.99, s
14	25.8, CH_3_	1.68, s	25.2, CH_3_	1.33, s	12.8, CH_3_	1.01, d (6.8)
15			66.9, CH_2_	3.89, br s	171.3, qC	
7-OH				3.08, s		

^a^ NMR data were measured in CDCl_3_; ^b^ NMR data were measured in acetone-*d*_6_.

**Table 2:** ^1^H (400 MHz) and ^13^C NMR (100 MHz) data of compounds **4**‒**6** in CDCl_3_.

Position	4		5		6	
	δ_C_, Type	δ_H_, Mult. (*J* in Hz)	δ_C_, Type	δ_H_, Mult. (*J* in Hz)	δ_C_, Type	δ_H_, Mult. (*J* in Hz)
1	29.9, CH_2_	2.17, m	30.1, CH_2_	2.27, d (20.0)	38.9, CH_2_	2.70, d (14.4)
		2.40, m		2.43, d (20.0)		2.79, d (14.4)
2	142.7, CH	7.12, br s	142.4, CH	7.26, br s	215.8, qC	
3	129.1, qC		130.6, qC		75.9, qC	
4	22.6, CH_2_	2.27, m	22.3, CH_2_	2.10, m	33.3, CH_2_	1.76, m
		2.35, m		2.58, d (18.0)		2.24, m
5	27.5, CH_2_	1.50, m	30.7, CH_2_	1.88, m	23.5, CH_2_	1.54, m
		1.82, m				1.80, m
6	39.5, qC		43.0, qC		55.2, qC	
7	36.8, CH	1.95, m	143.8, qC		81.8, qC	
8	70.8, CH	3.91, dd (12.0, 6.8)	135.9, qC		213.8, qC	
9	27.7, CH_2_	1.63,m	192.3, qC		33.7, CH_2_	2.41,m
						2.74, m
10	35.6, CH_2_	1.48, m	47.1, CH_2_	2.22, d (18.0)	36.5, CH_2_	1.61, m
				2.78, d (18.0)		1.91, m
11	36.6, qC		40.8, qC		39.0, qC	
12	25.7, CH_3_	0.84, s	23.8, CH_3_	1.01, s	25.0, CH_3_	1.28, s
13	26.3, CH_3_	0.95, s	24.8, CH_3_	1.05, s	28.6, CH_3_	1.00, s
14	11.4, CH_3_	0.98, d (6.8)	15.1, CH_3_	1.85, s	25.4, CH_3_	1.42, s
15	171.4, qC		170.9, qC		67.1, CH_2_	3.48, d (11.6)
						3.85, d (11.6)
7-OH						4.15, br s

## Supplementary Materials

The following are available online at www.mdpi.com/1660-3397/14/7/132/s1, NMR spectra of compounds **1**–**6** and CIF files for compounds **1** and **2**.
