# Supplementary material for: Chamigrane Sesquiterpenes from a Basidiomycetous Endophytic Fungus XG8D Associated with Thai Mangrove Xylocarpus granatum
Source: Mar Drugs. 2016 Jul 15;14(7):132. doi: 10.3390/md14070132 (PMC4962022; doi:10.3390/md14070132)
Supplement: Supplementary file 1 [file marinedrugs-14-00132-s001.pdf]

Supplementary Materials: Chamigrane Sesquiterpenes from a Basidiomycetous Endophytic Fungus XG8D Associated with Thai Mangrove *Xylocarpus granatum*

Siwattra Choodej, Thapong Teerawatananond, Tohru Mitsunaga and Khanitha Pudhom

Figure	Description	Page
**Figure S1**	^{1}H NMR spectrum ($CDCl_3$) of Merulinol A (**1**)	S2
**Figure S2**	^{13}C NMR spectrum ($CDCl_3$) of Merulinol A (**1**)	S2
**Figure S3**	^{1}H-^{1}H COSY spectrum ($CDCl_3$) of Merulinol A (**1**)	S3
**Figure S4**	HSQC spectrum ($CDCl_3$) of Merulinol A (**1**)	S3
**Figure S5**	HMBC spectrum ($CDCl_3$) of Merulinol A (**1**)	S4
**Figure S6**	^{1}H NMR spectrum (acetone-d_6) of Merulinol B (**2**)	S4
**Figure S7**	^{13}C NMR spectrum (acetone-d_6) of Merulinol B (**2**)	S5
**Figure S8**	^{1}H-^{1}H COSY spectrum (acetone-d_6) of Merulinol B (**2**)	S5
**Figure S9**	HSQC spectrum (acetone-d_6) of Merulinol B (**2**)	S6
**Figure S10**	HMBC spectrum (acetone-d_6) of Merulinol B (**2**)	S6
**Figure S11**	NOESY spectrum (acetone-d_6) of Merulinol B (**2**)	S7
**Figure S12**	^{1}H NMR spectrum ($CDCl_3$) of Merulinol C (**3**)	S7
**Figure S13**	^{13}C NMR spectrum ($CDCl_3$) of Merulinol C (**3**)	S8
**Figure S14**	^{1}H-^{1}H COSY spectrum ($CDCl_3$) of Merulinol C (**3**)	S8
**Figure S15**	HSQC spectrum ($CDCl_3$) of Merulinol C (**3**)	S9
**Figure S16**	HMBC spectrum ($CDCl_3$) of Merulinol C (**3**)	S9
**Figure S17**	NOESY spectrum ($CDCl_3$) of Merulinol C (**3**)	S10
**Figure S18**	^{1}H NMR spectrum ($CDCl_3$) of Merulinol D (**4**)	S10
**Figure S19**	^{13}C NMR spectrum ($CDCl_3$) of Merulinol D (**4**)	S11
**Figure S20**	^{1}H-^{1}H COSY spectrum ($CDCl_3$) of Merulinol D (**4**)	S11
**Figure S21**	HSQC spectrum ($CDCl_3$) of Merulinol D (**4**)	S12
**Figure S22**	HMBC spectrum ($CDCl_3$) of Merulinol D (**4**)	S12
**Figure S23**	NOESY spectrum ($CDCl_3$) of Merulinol D (**4**)	S13
**Figure S24**	^{1}H NMR spectrum ($CDCl_3$) of Merulinol E (**5**)	S13
**Figure S25**	^{13}C NMR spectrum ($CDCl_3$) of Merulinol E (**5**)	S14
**Figure S26**	^{1}H-^{1}H COSY spectrum ($CDCl_3$) of Merulinol E (**5**)	S14
**Figure S27**	HSQC spectrum ($CDCl_3$) of Merulinol E (**5**)	S15
**Figure S28**	HMBC spectrum ($CDCl_3$) of Merulinol E (**5**)	S15
**Figure S29**	NOESY spectrum ($CDCl_3$) of Merulinol E (**5**)	S16
**Figure S30**	^{1}H NMR spectrum ($CDCl_3$) of Merulinol F (**6**)	S16
**Figure S31**	^{13}C NMR spectrum ($CDCl_3$) of Merulinol F (**6**)	S17
**Figure S32**	^{1}H-^{1}H COSY spectrum ($CDCl_3$) of Merulinol F (6)	S17
**Figure S33**	HSQC spectrum ($CDCl_3$) of Merulinol F (**6**)	S18
**Figure S34**	HMBC spectrum ($CDCl_3$) of Merulinol F (**6**)	S18

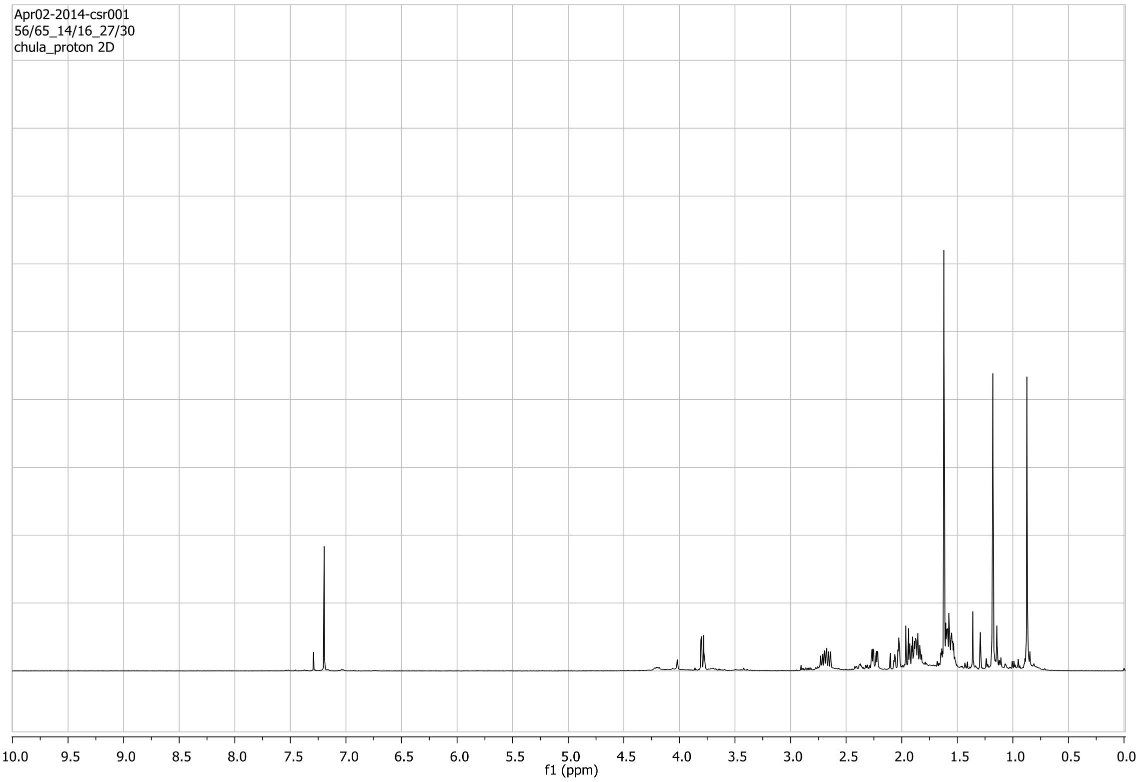

Figure S1. ^{1}H NMR spectrum ($CDCl_3$) of Merulinol A (**1**).

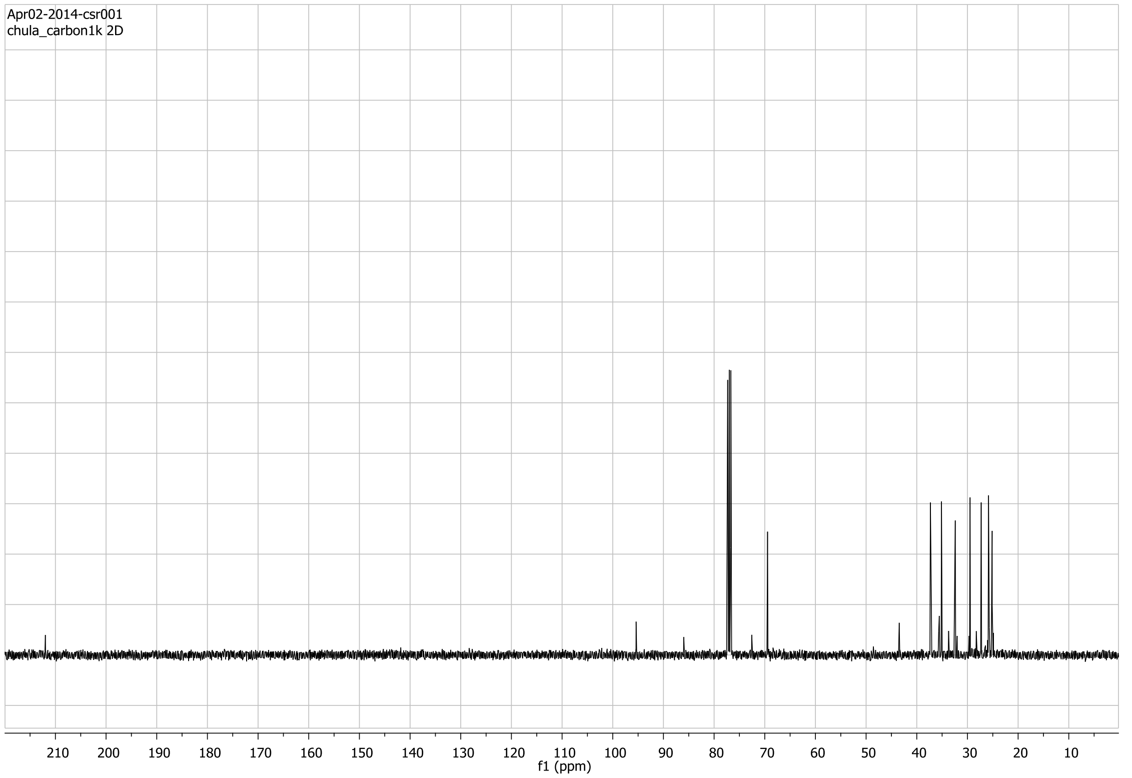

Figure S2. ^{13}C NMR spectrum ($CDCl_3$) of Merulinol A (**1**).

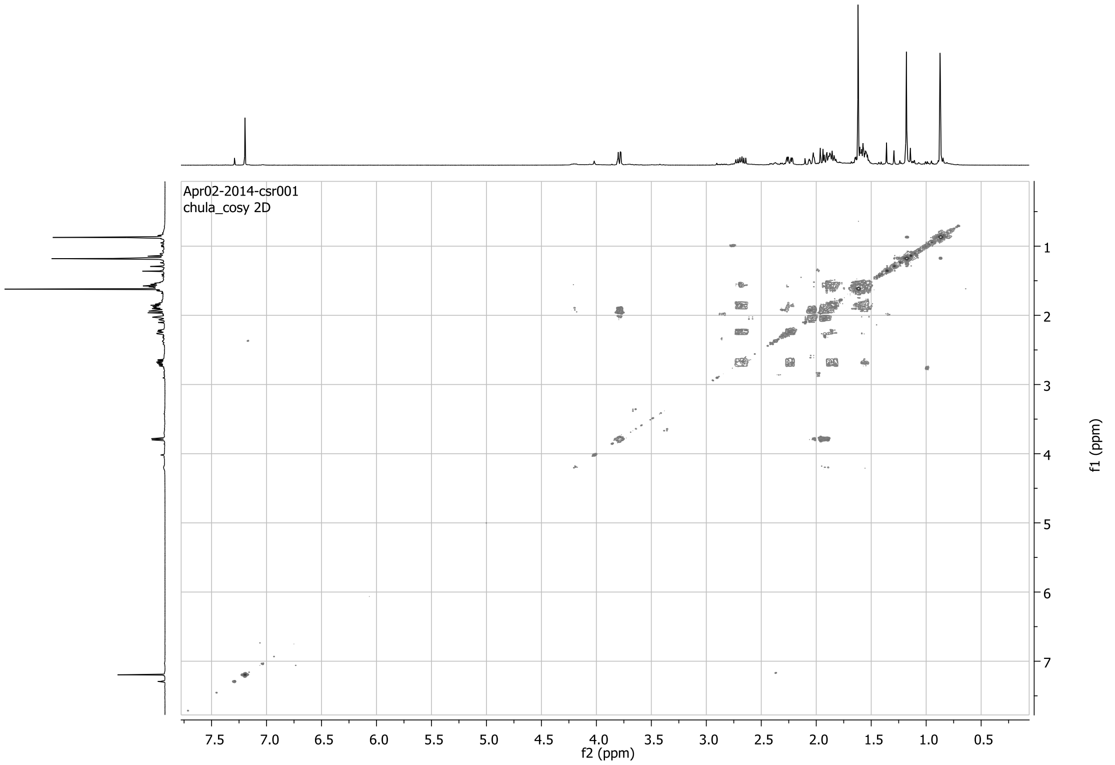

Figure S3. ^{1}H-^{1}H COSY spectrum ($CDCl_3$) of Merulinol A (**1**).

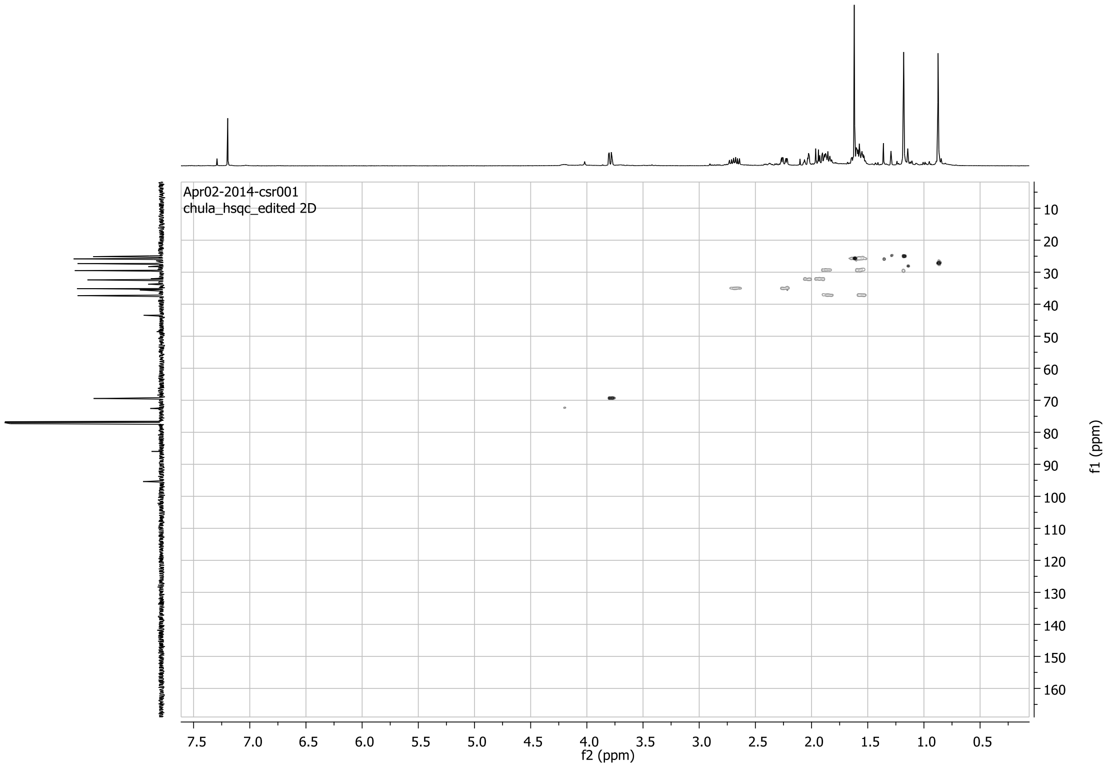

Figure S4. HSQC spectrum ($CDCl_3$) of Merulinol A (**1**).

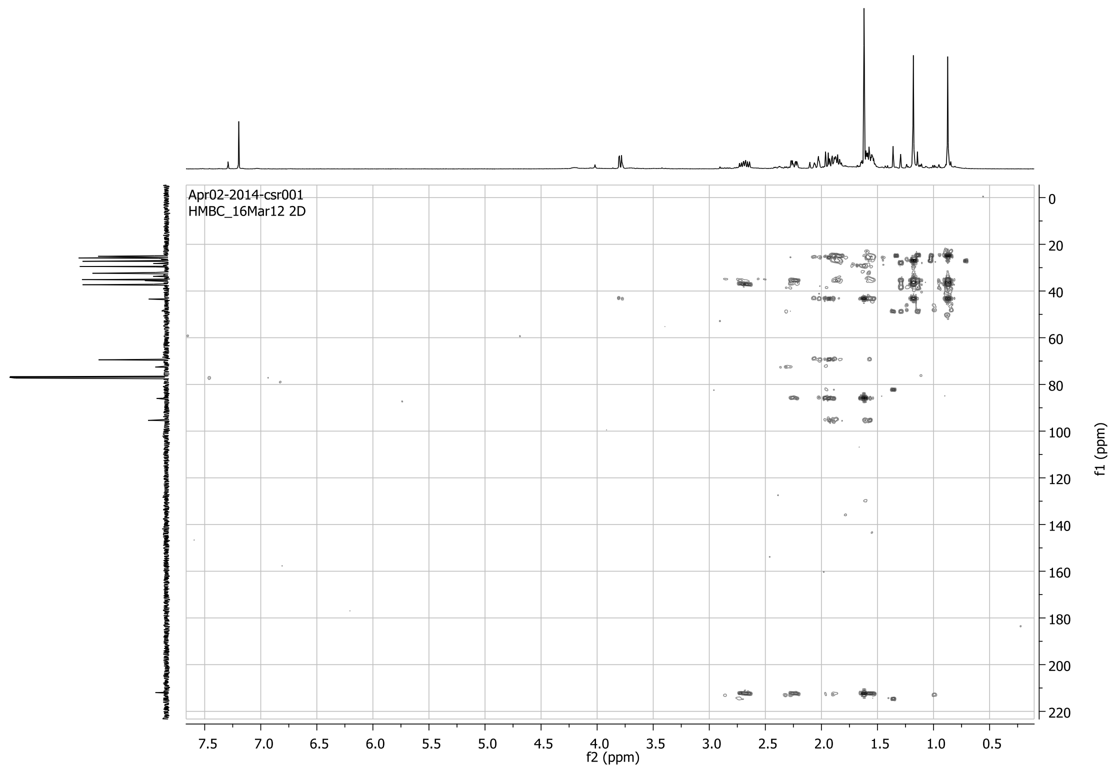

Figure S5. HMBC spectrum ($CDCl_3$) of Merulinol A (**1**).

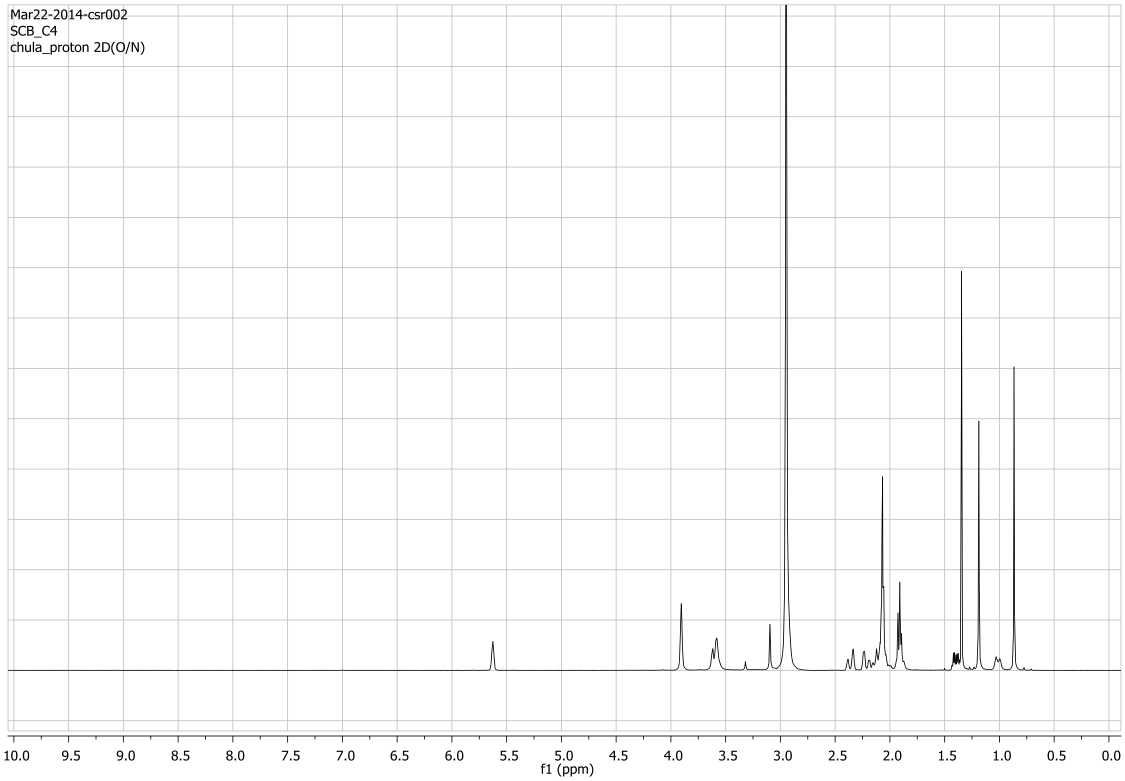

Figure S6. ^1H NMR spectrum (acetone-d_6) of Merulinol B (**2**).

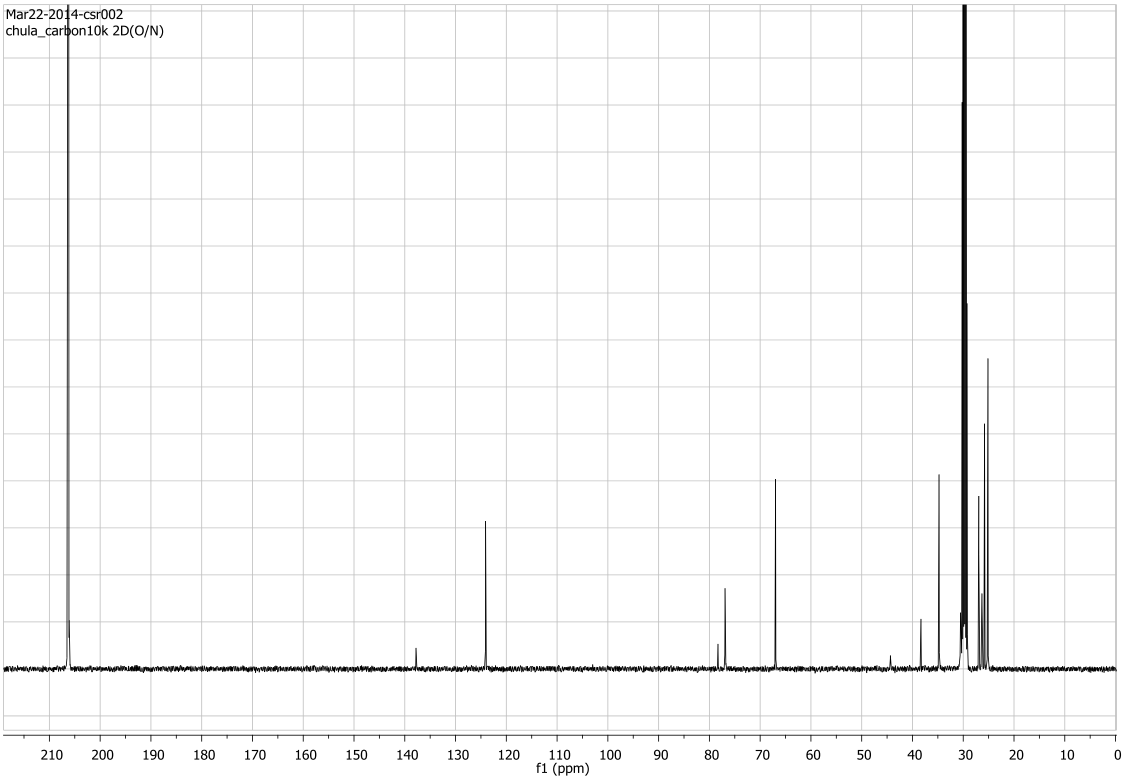

Figure S7. ^{13}C NMR spectrum (acetone-d_6) of Merulinol B (**2**).

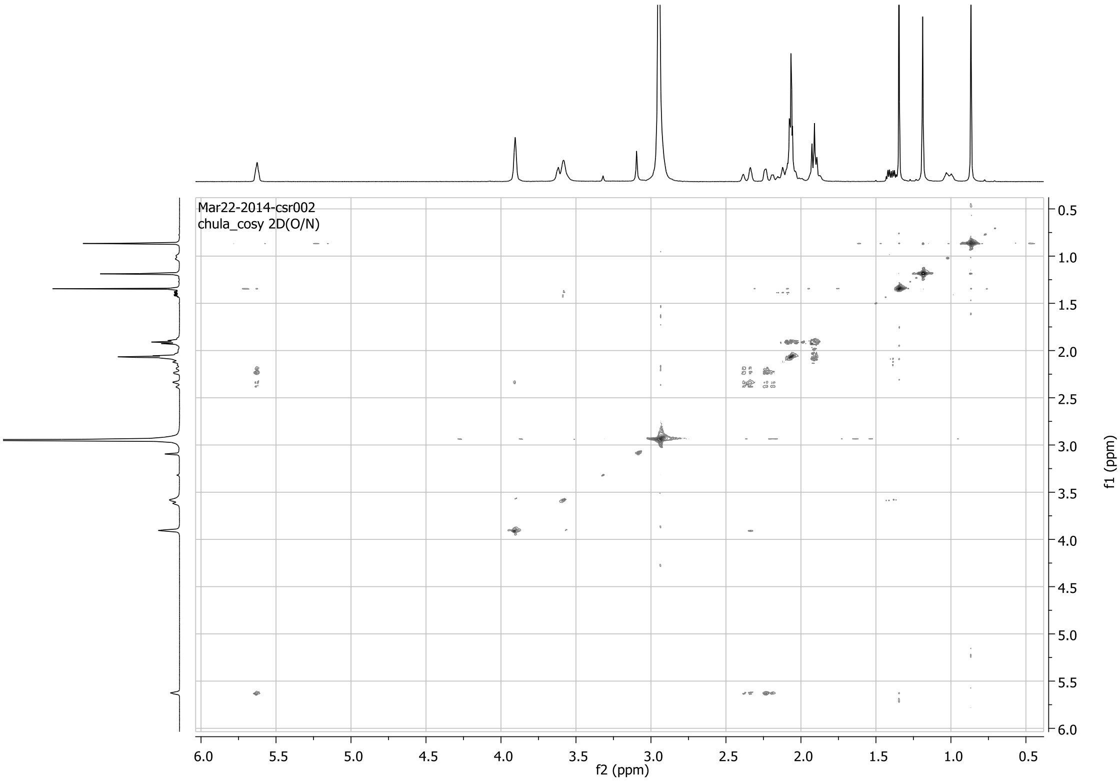

Figure S8. ^{1}H-^{1}H COSY spectrum (acetone-d_6) of Merulinol B (**2**).

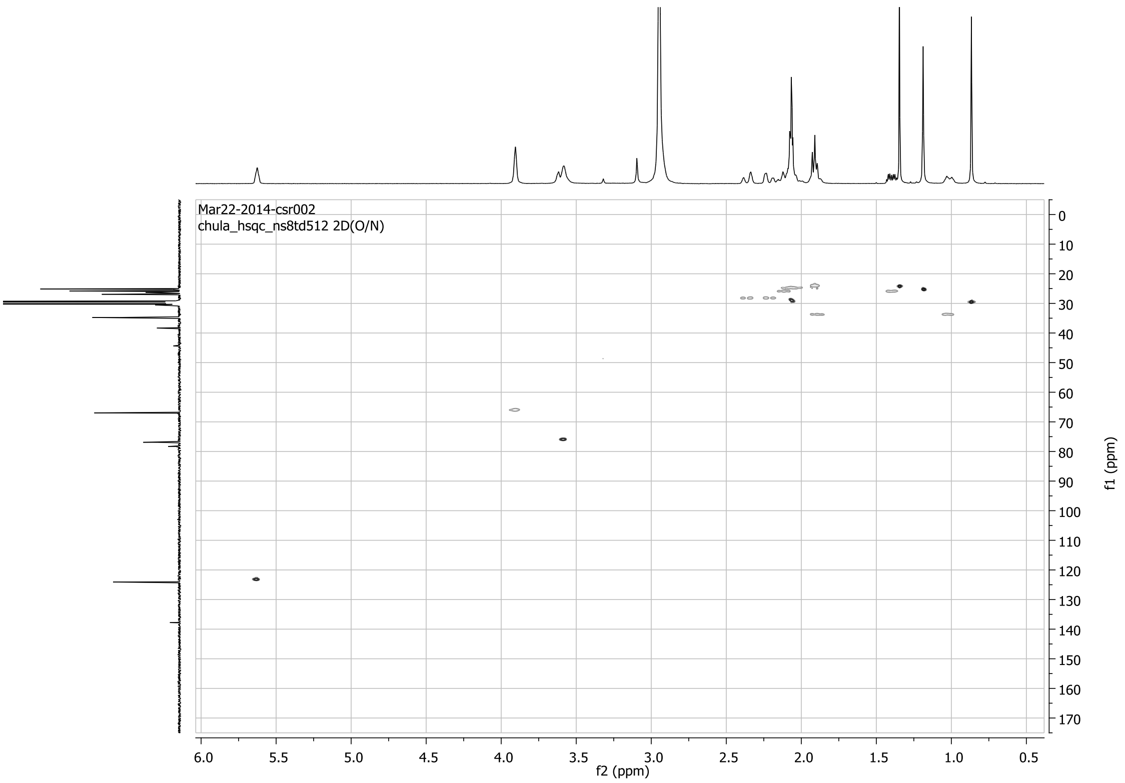

Figure S9. HSQC spectrum (acetone-d_6) of Merulinol B (**2**).

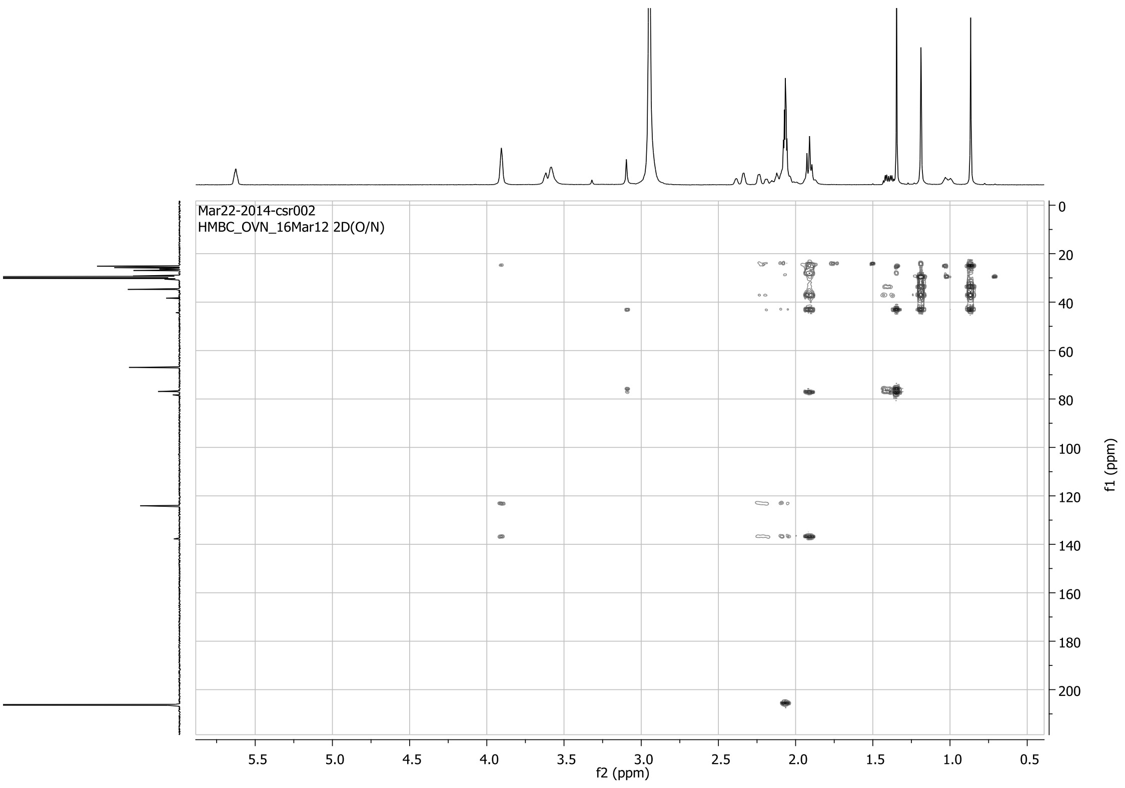

Figure S10. HMBC spectrum (acetone-d_6) of Merulinol B (**2**).

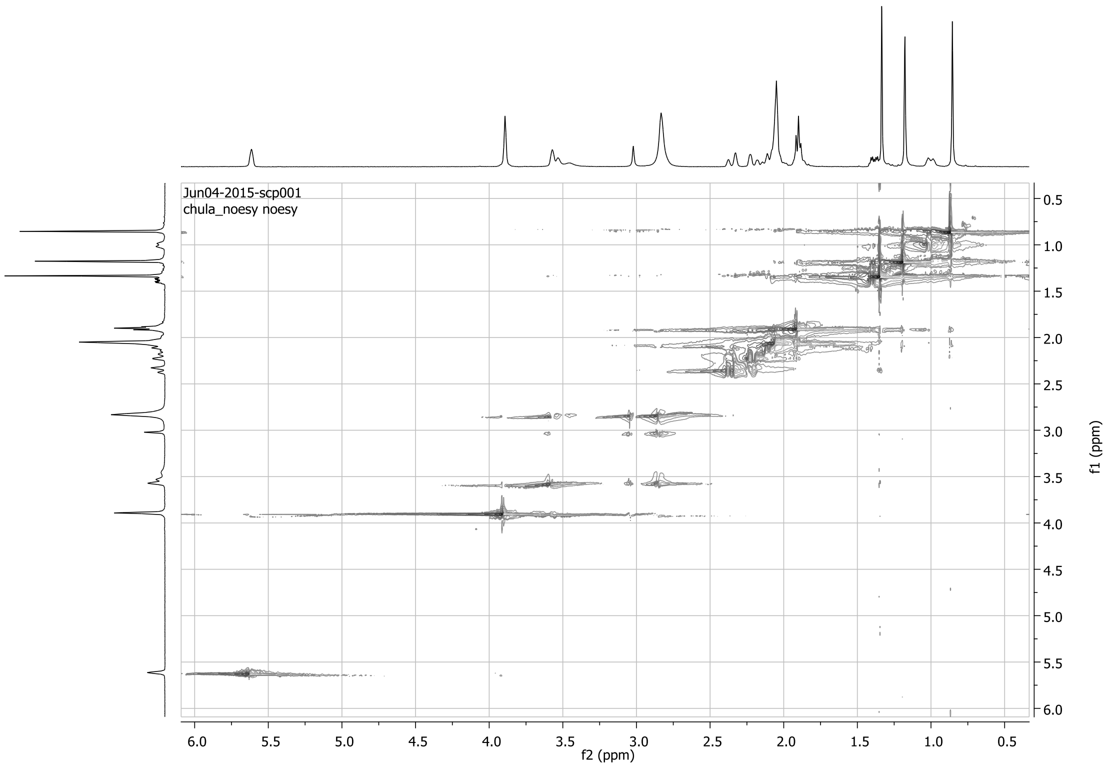

Figure S11. NOESY spectrum (acetone-d_6) of Merulinol B (**2**).

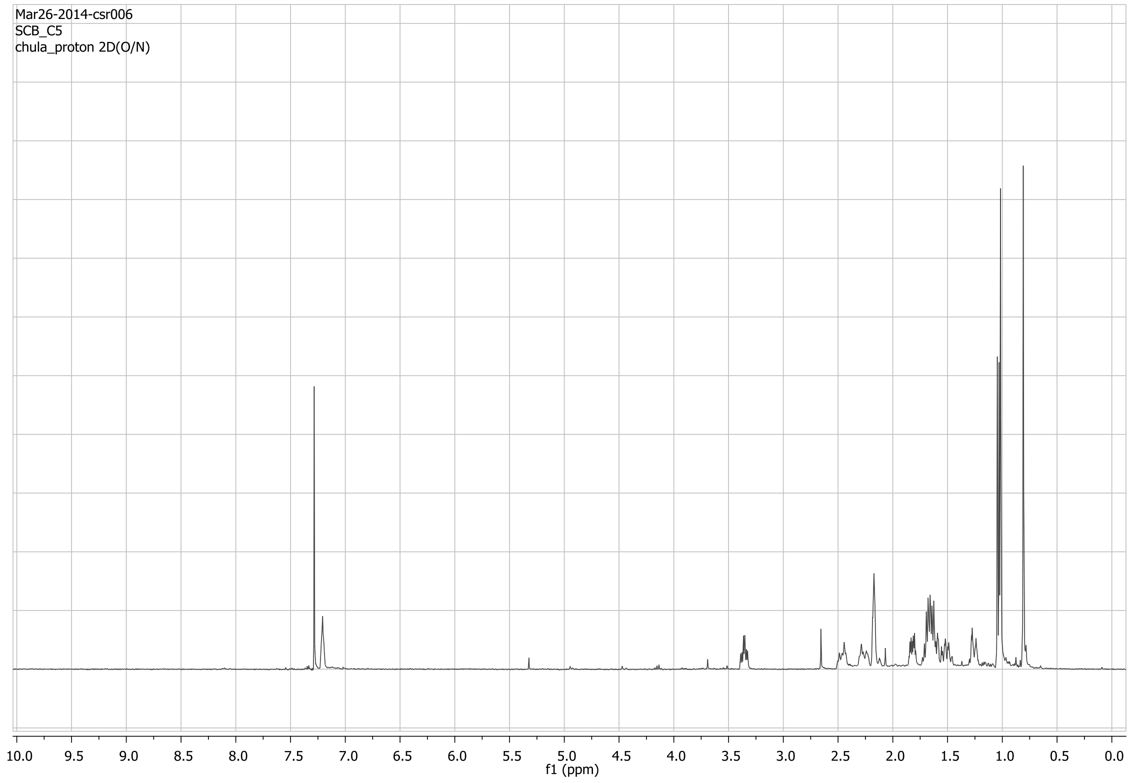

Figure S12. ^{1}H NMR spectrum ($CDCl_3$) of Merulinol C (**3**).

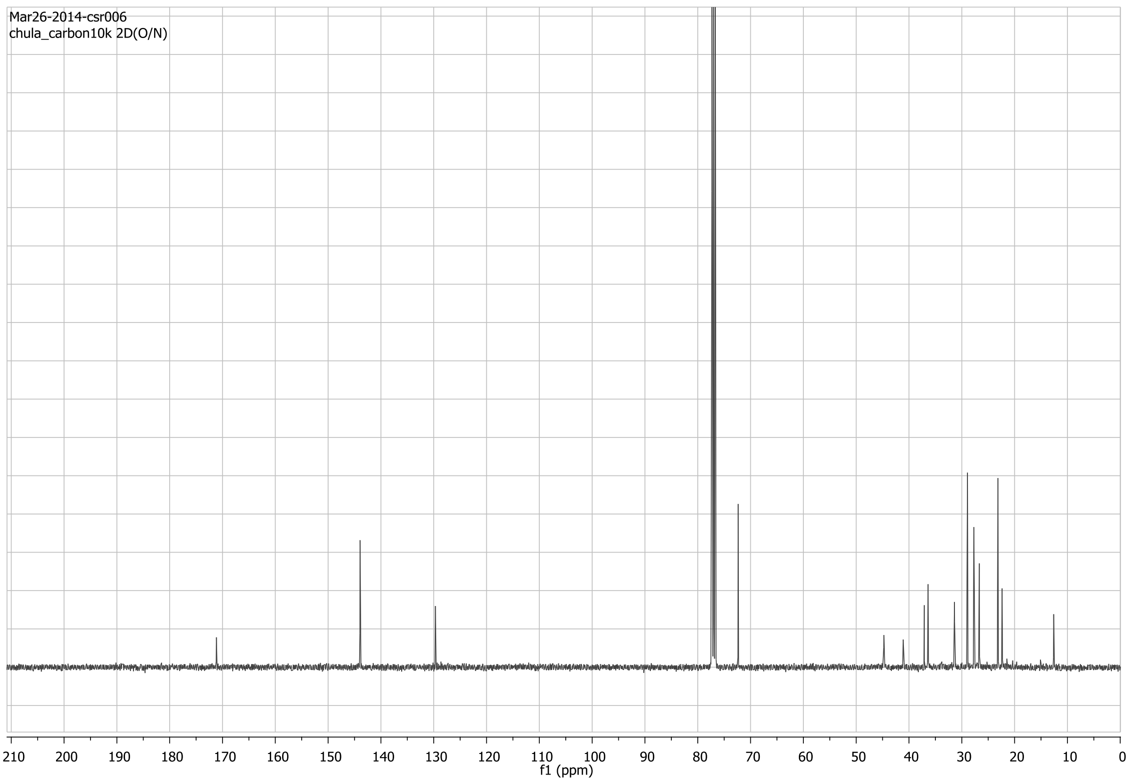

Figure S13. ^{13}C NMR spectrum ($CDCl_3$) of Merulinol C (**3**).

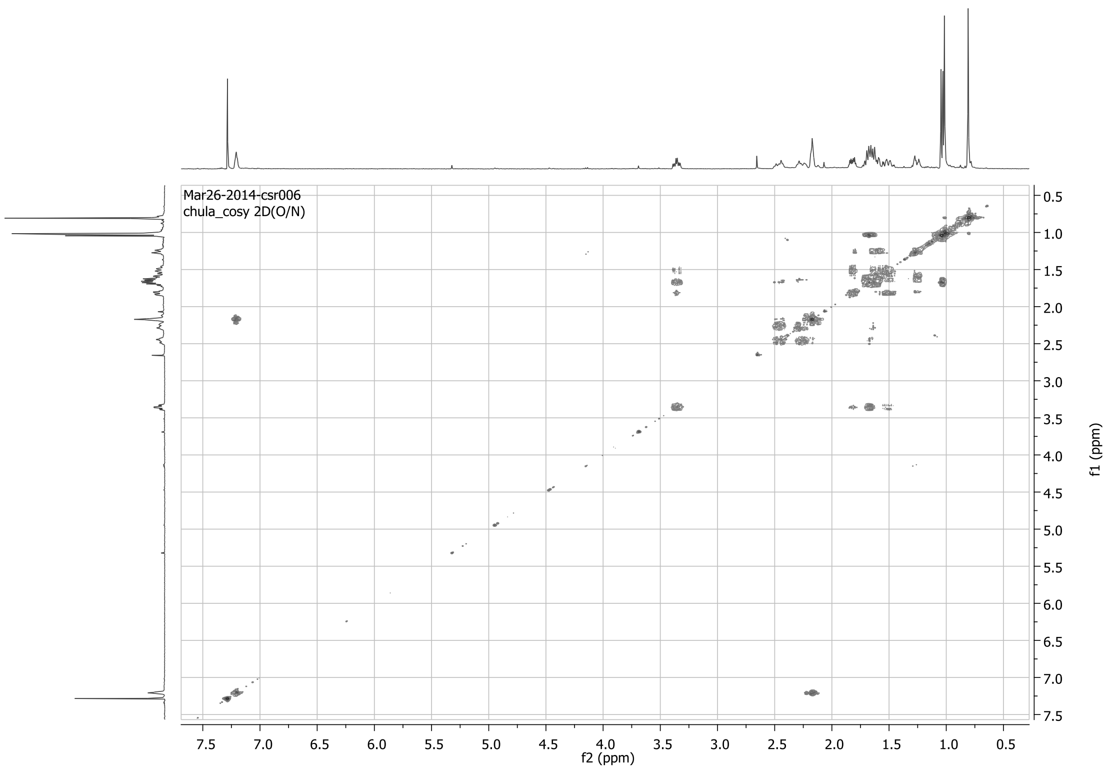

Figure S14. ^{1}H-^{1}H COSY spectrum ($CDCl_3$) of Merulinol C (**3**).

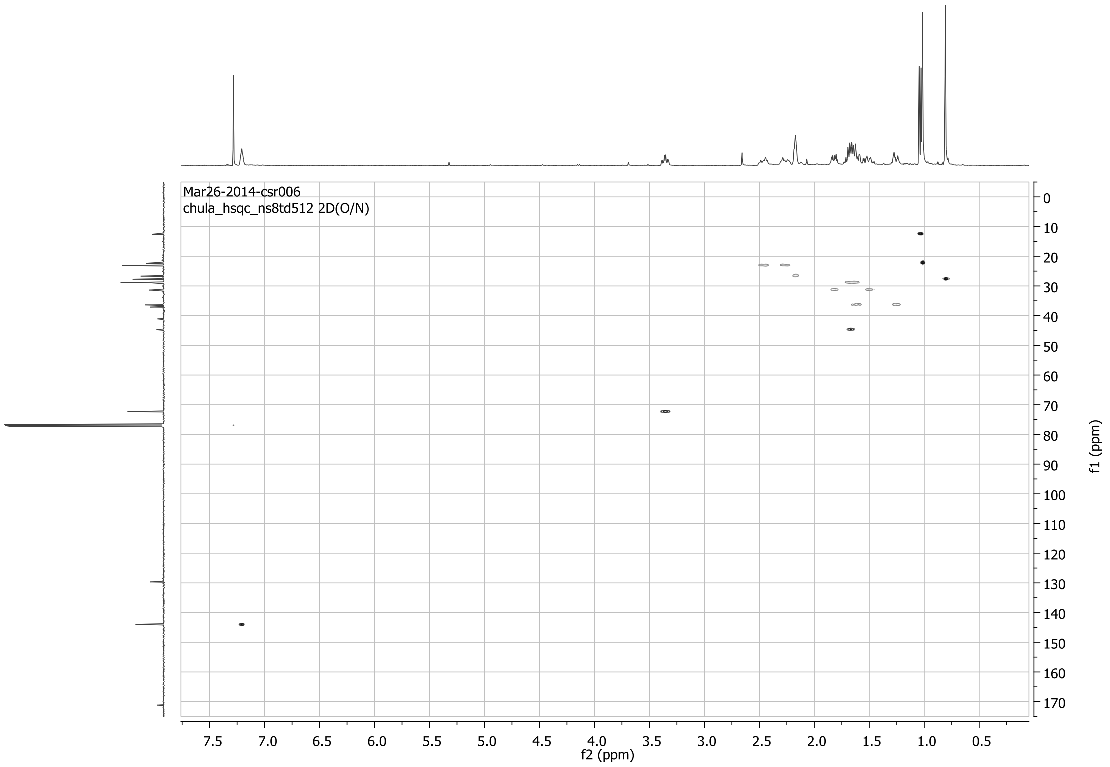

Figure S15. HSQC spectrum ($CDCl_3$) of Merulinol C (**3**).

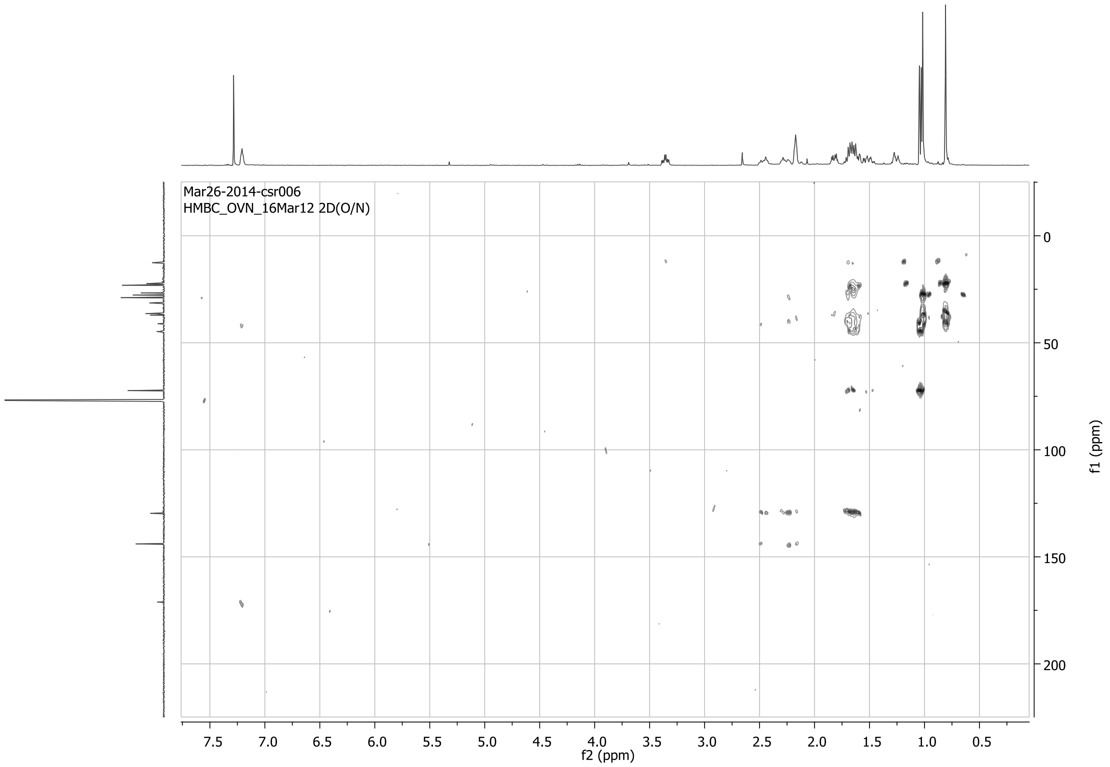

Figure S16. HMBC spectrum ($CDCl_3$) of Merulinol C (**3**).

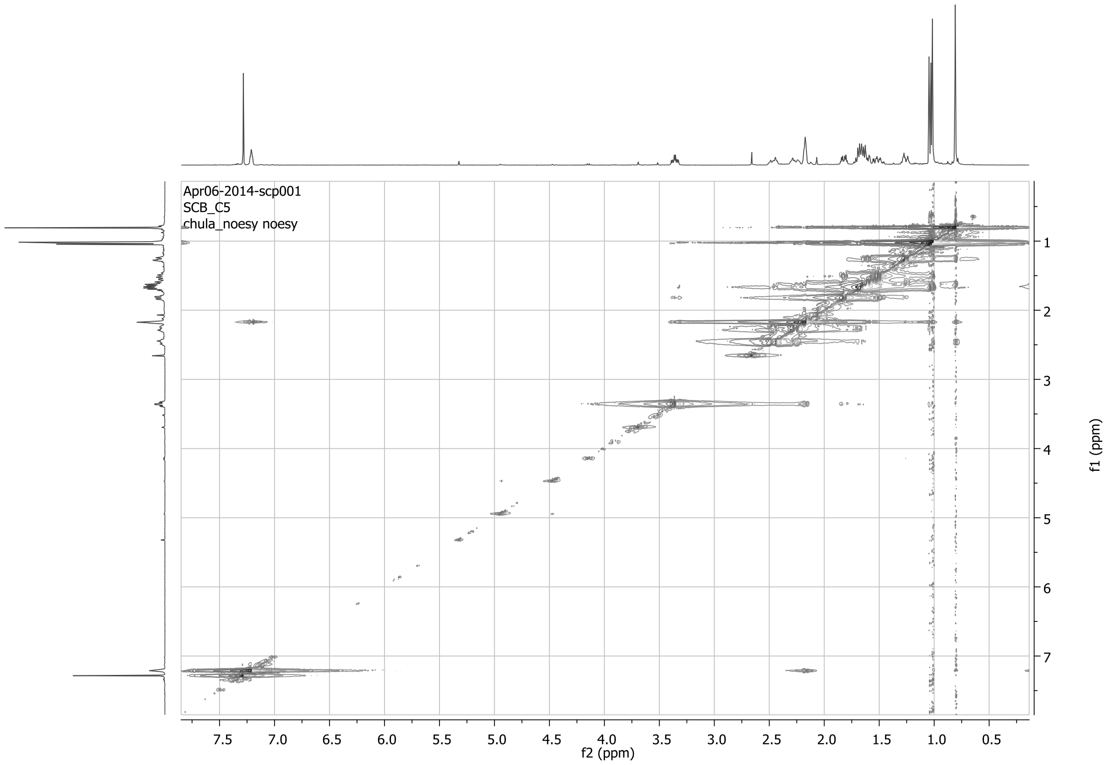

Figure S17. NOESY spectrum ($CDCl_3$) of Merulinol C (**3**).

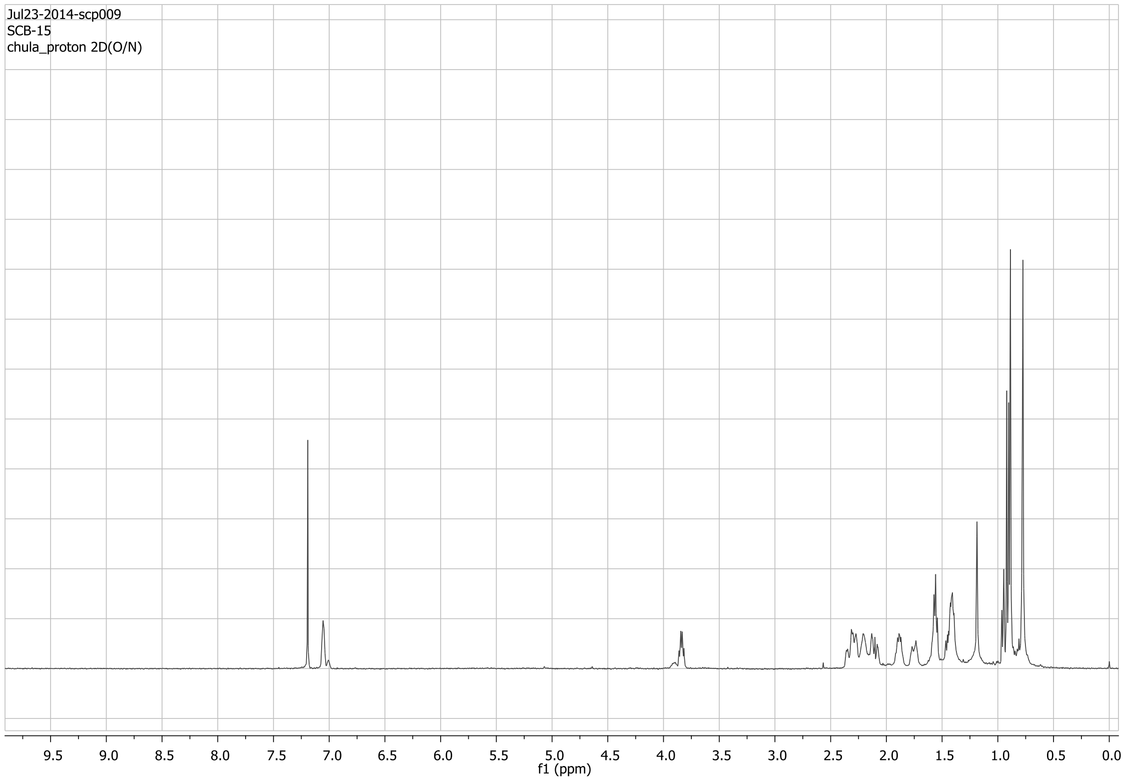

Figure S18. ^{1}H NMR spectrum ($CDCl_3$) of Merulinol D (**4**).

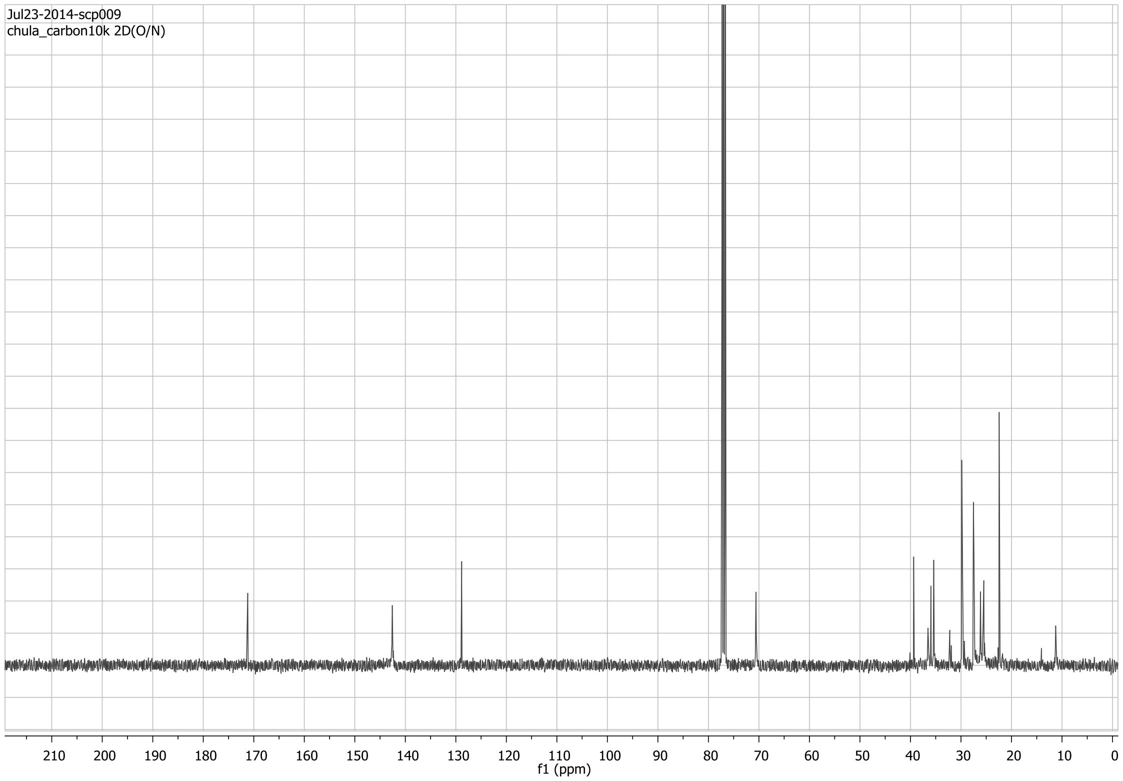

Figure S19. ^{13}C NMR spectrum ($CDCl_3$) of Merulinol D (**4**).

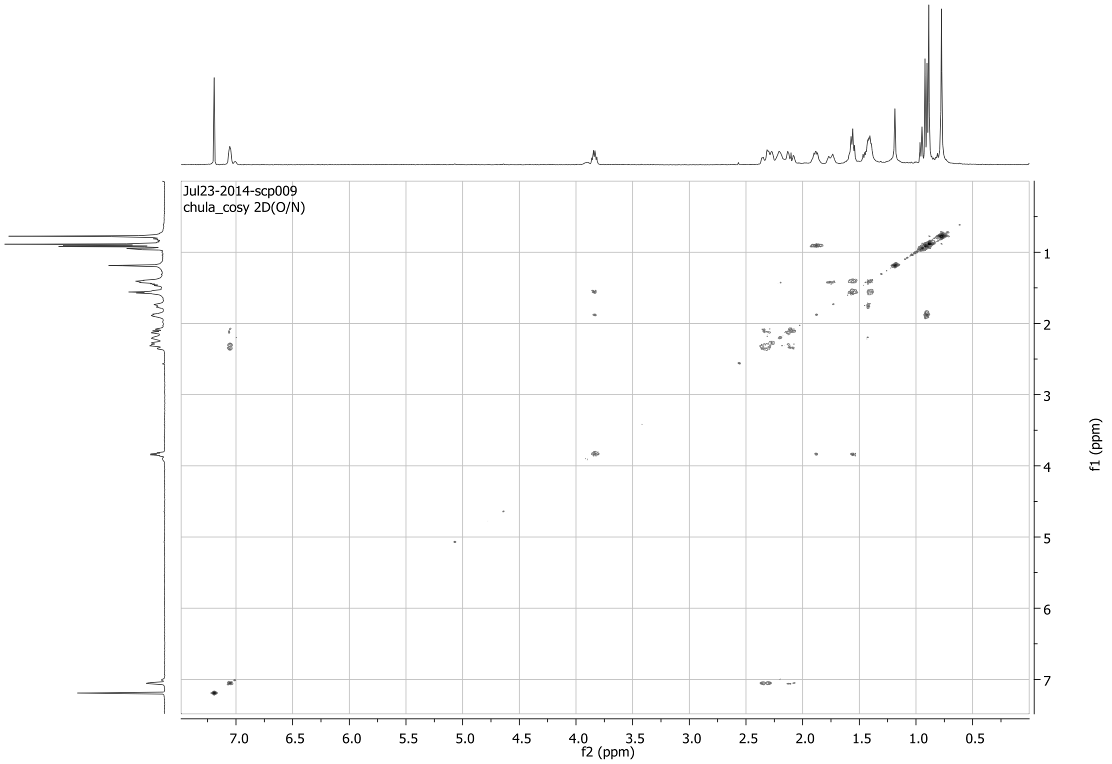

Figure S20. ^{1}H-^{1}H COSY spectrum ($CDCl_3$) of Merulinol D (**4**).

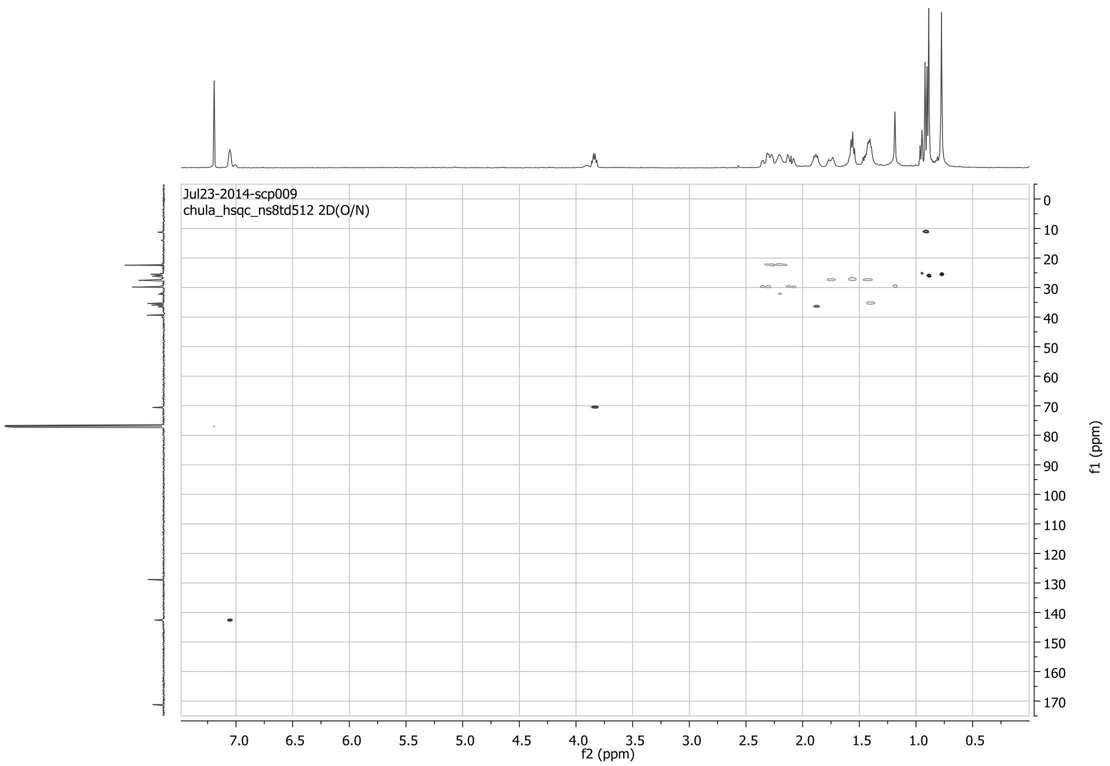

Figure S21. HSQC spectrum ($CDCl_3$) of Merulinol D (**4**).

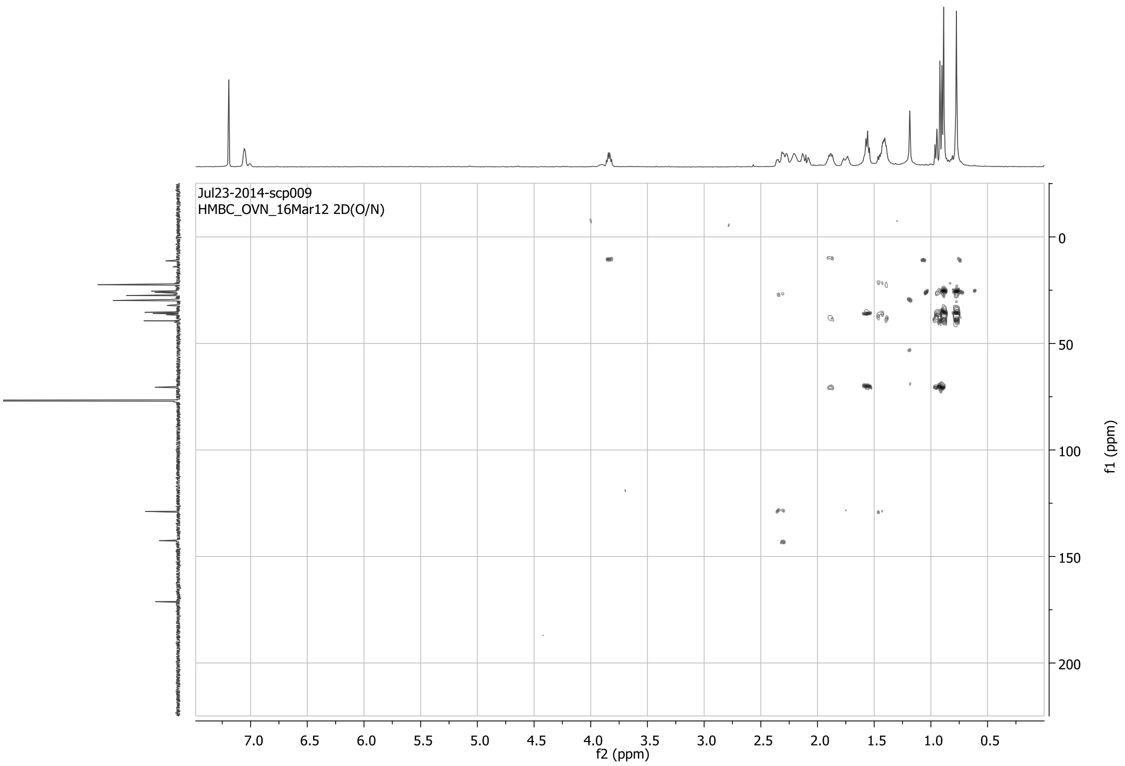

Figure S22. HMBC spectrum ($CDCl_3$) of Merulinol D (**4**).

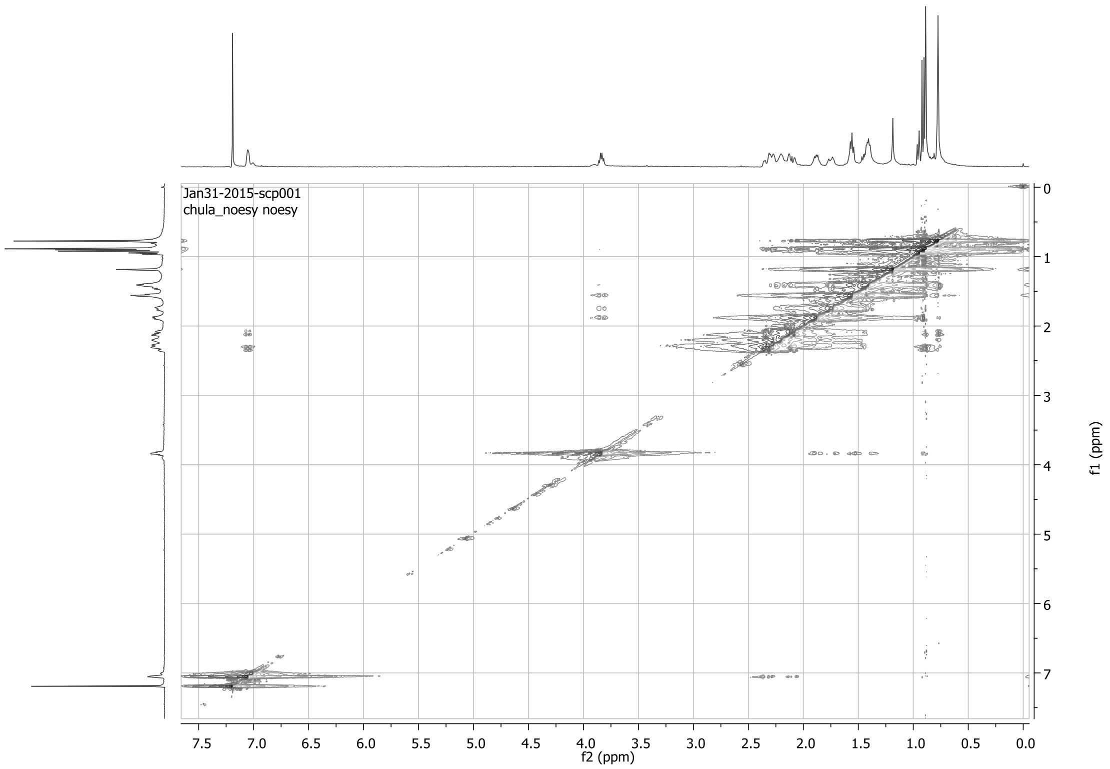

Figure S23. NOESY spectrum ($CDCl_3$) of Merulinol D (**4**).

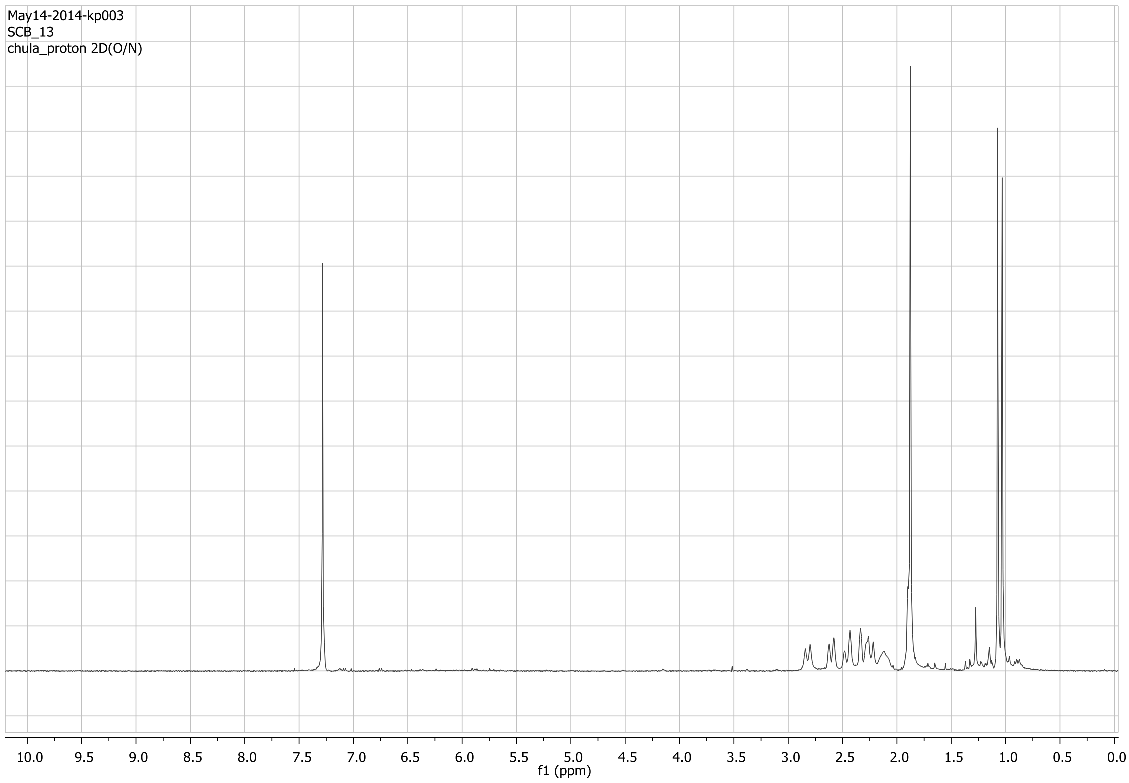

Figure S24. ^1H NMR spectrum ($CDCl_3$) of Merulinol E (**5**).

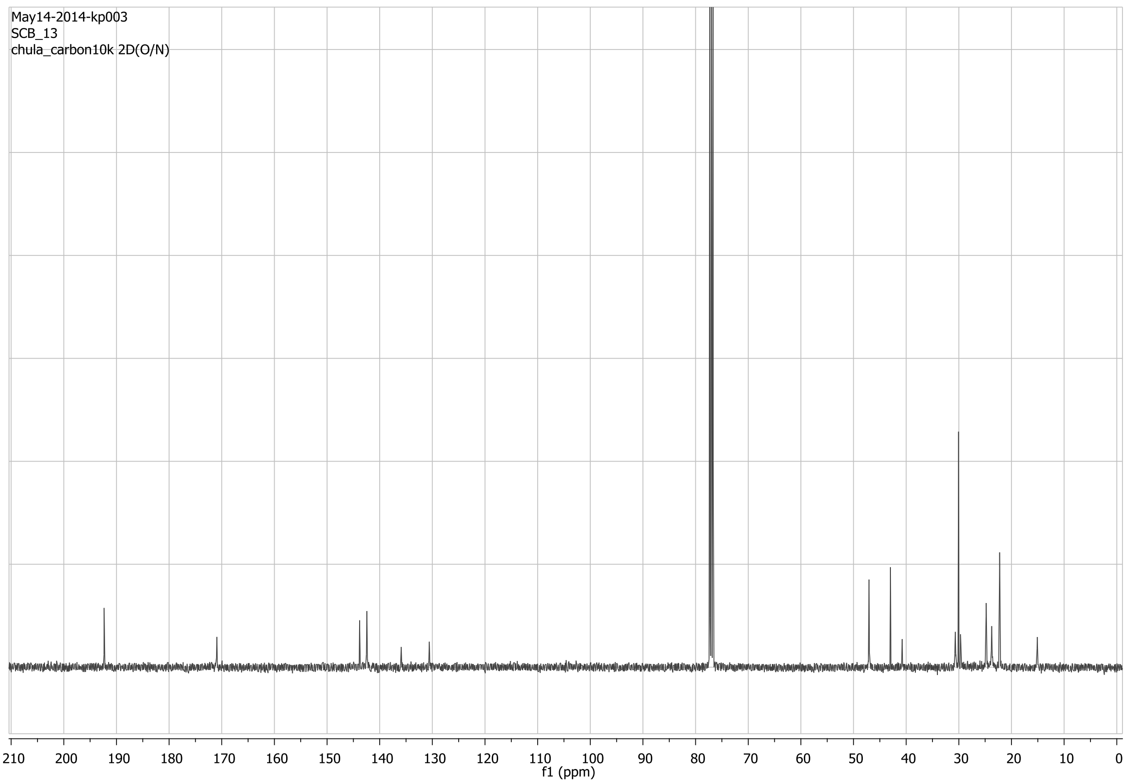

Figure S25. ^{13}C NMR spectrum ($CDCl_3$) of Merulinol E (**5**).

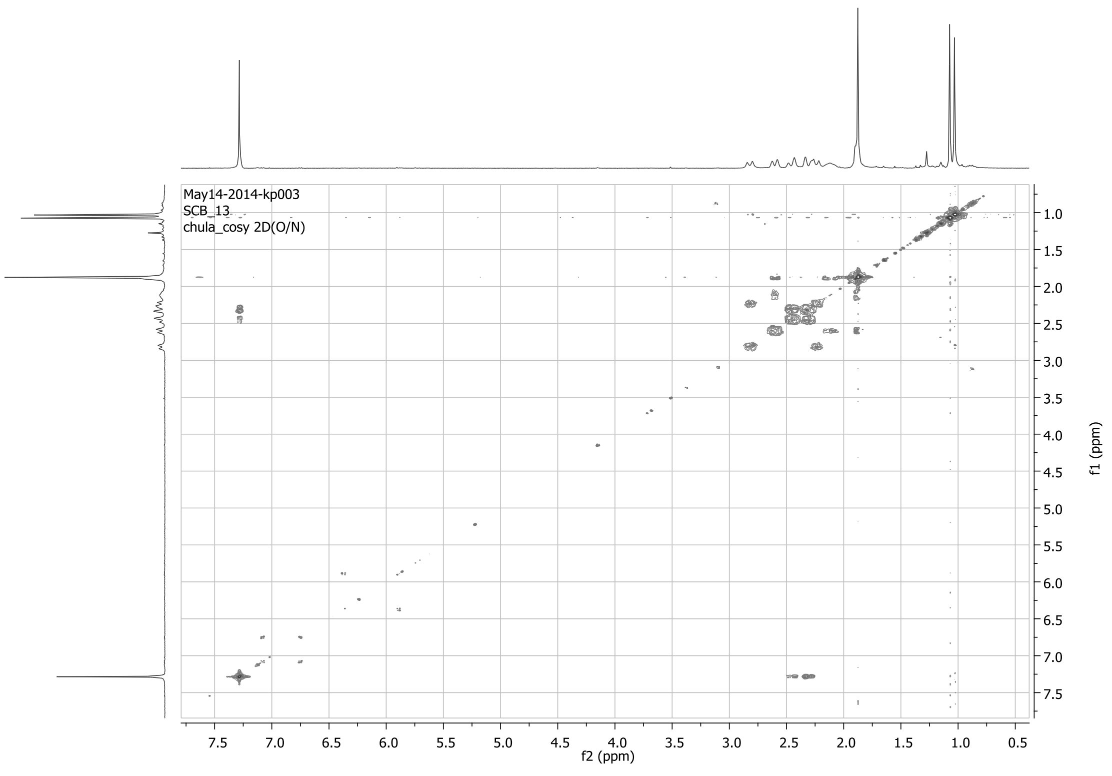

Figure S26. ^{1}H-^{1}H COSY spectrum ($CDCl_3$) of Merulinol E (**5**).

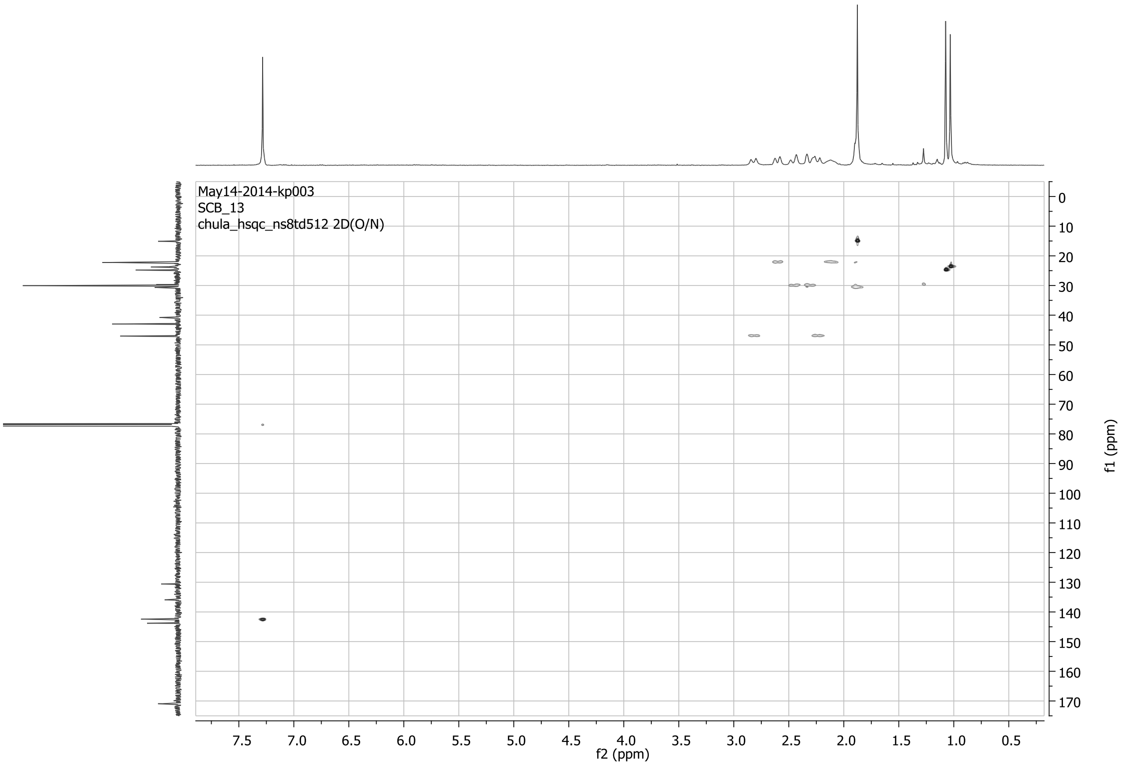

Figure S27. HSQC spectrum ($CDCl_3$) of Merulinol E (**5**).

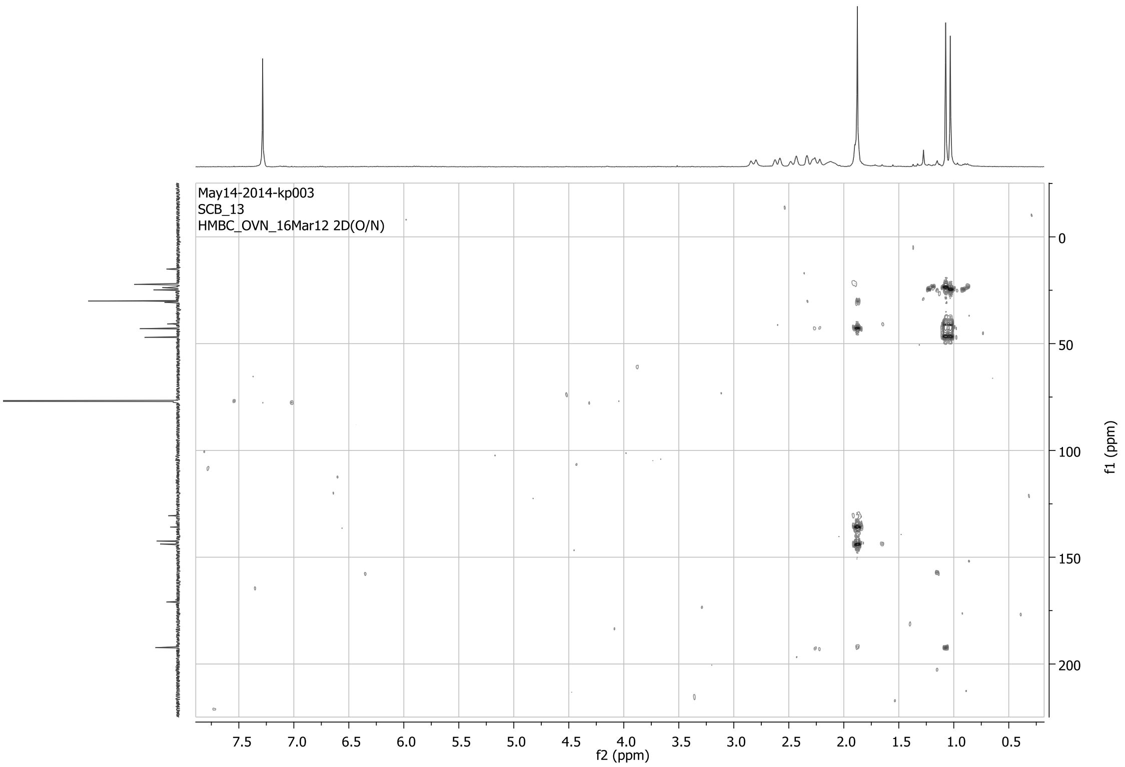

Figure S28. HMBC spectrum ($CDCl_3$) of Merulinol E (**5**).

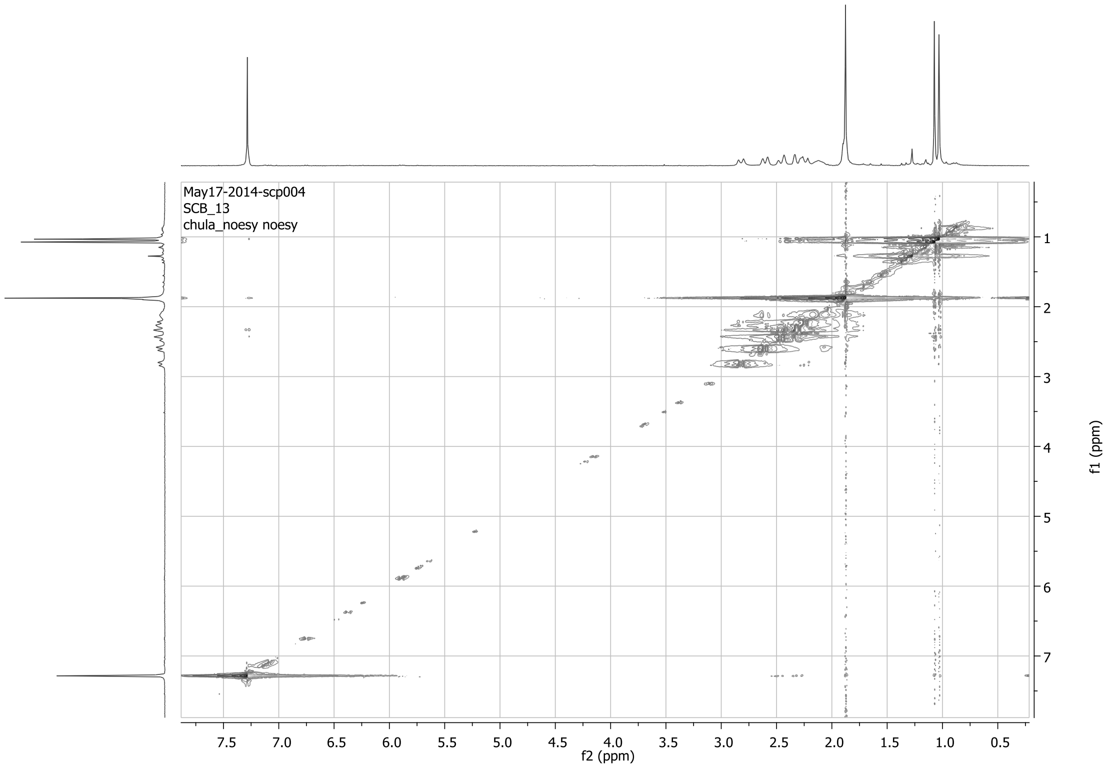

Figure S29. NOESY spectrum ($CDCl_3$) of Merulinol E (**5**).

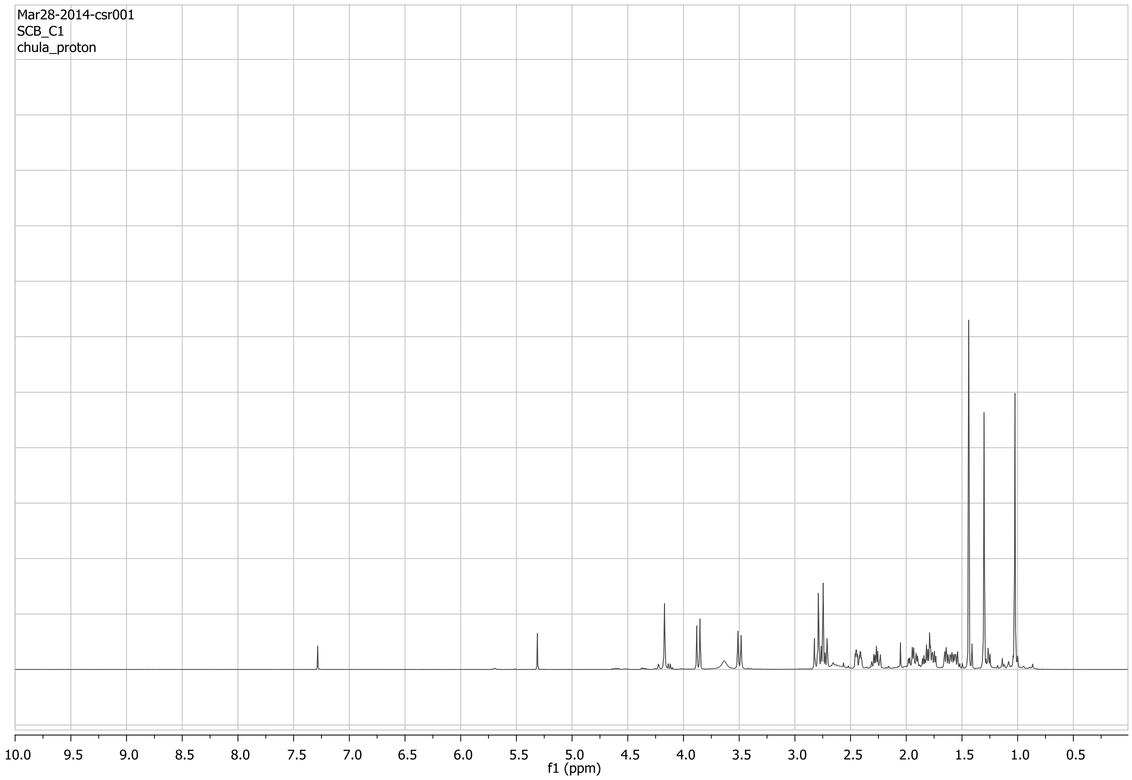

Figure S30. ^{1}H spectrum ($CDCl_3$) of Merulinol F (**6**).

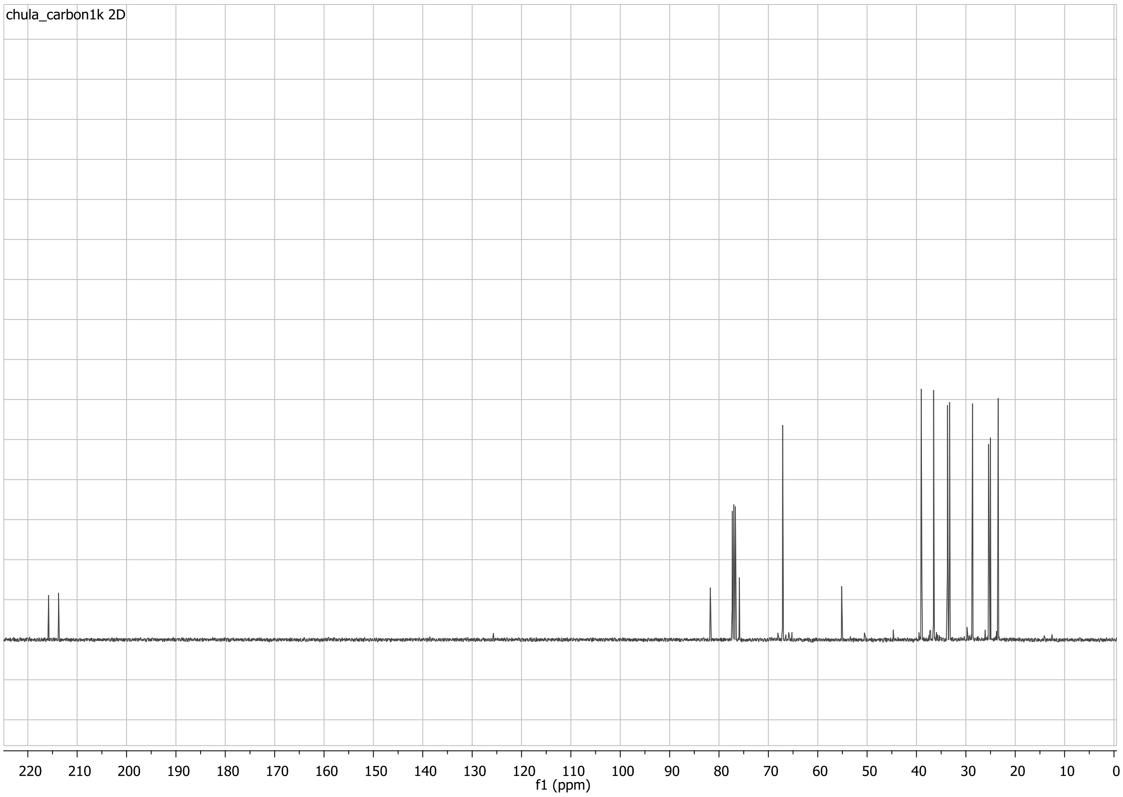

Figure S31. ^{13}C spectrum ($CDCl_3$) of Merulinol F (**6**).

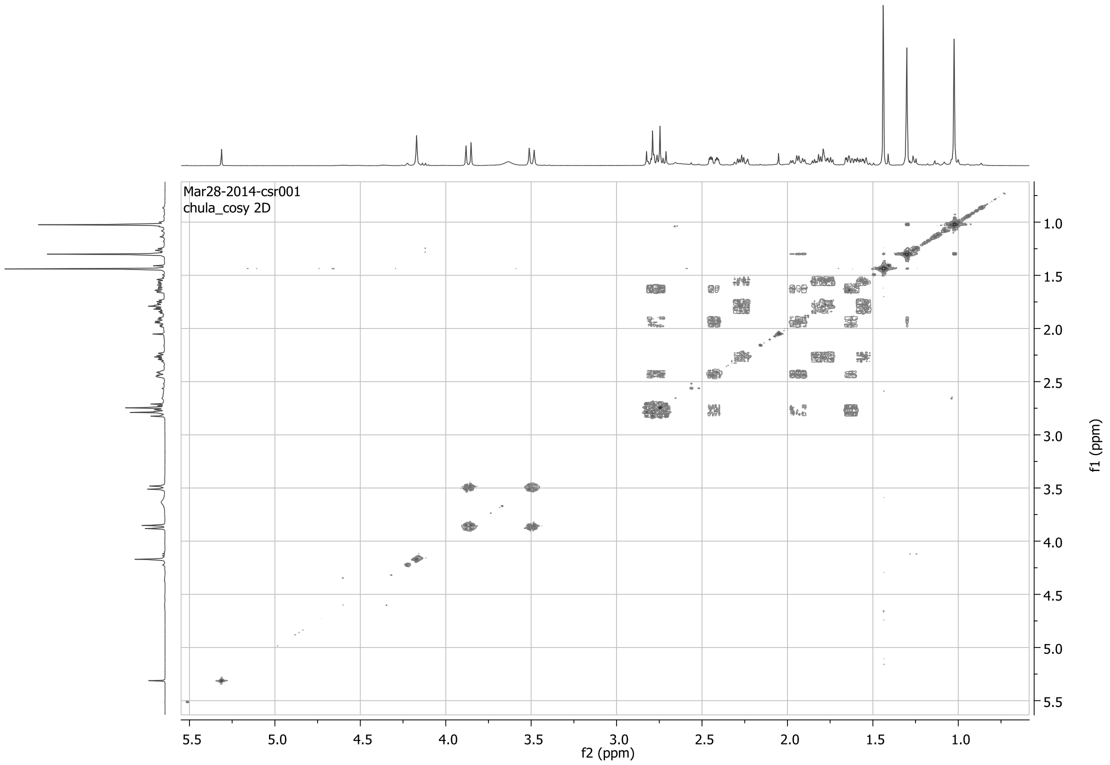

Figure S32. ^{1}H-^{1}H COSY spectrum ($CDCl_3$) of Merulinol F (**6**).

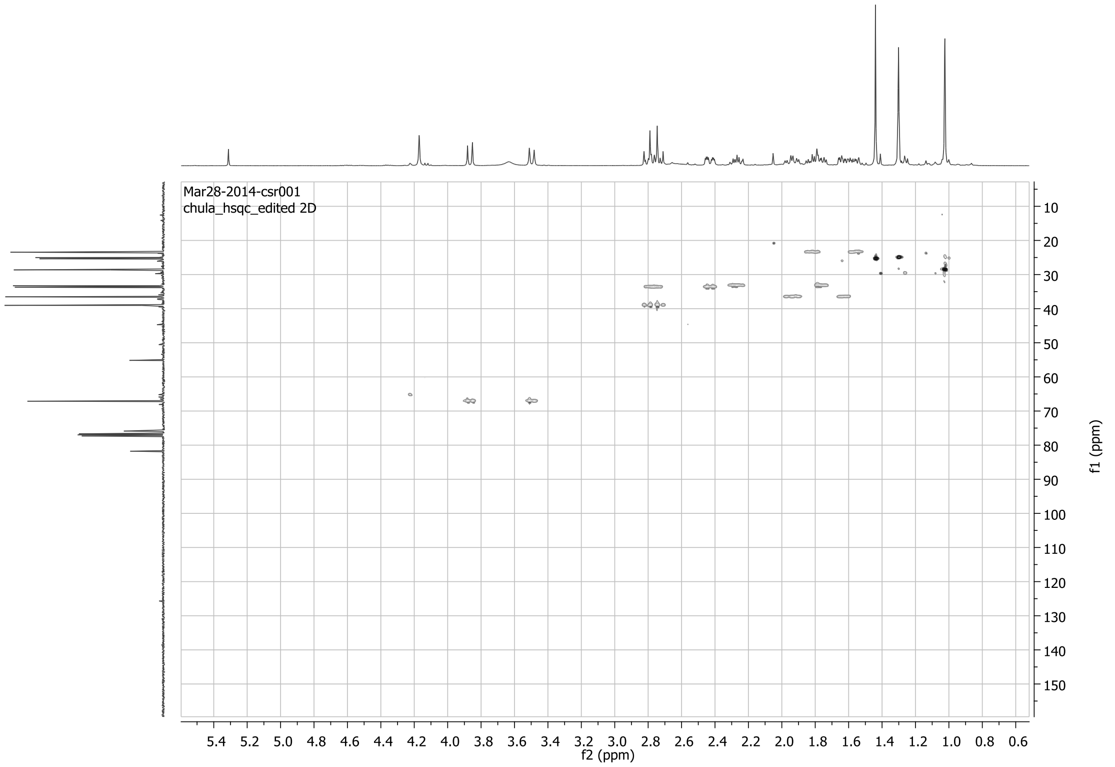

Figure S33. HSQC spectrum ($CDCl_3$) of Merulinol F (**6**).

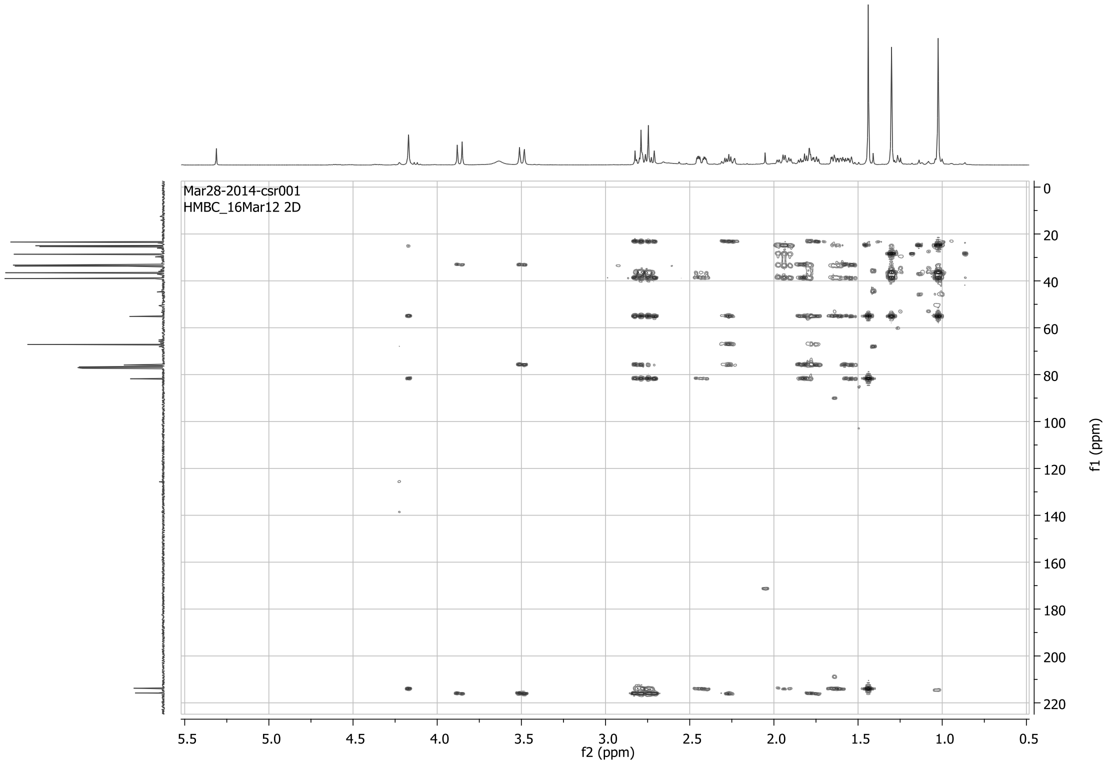

Figure S34. HMBC spectrum ($CDCl_3$) of Merulinol F (**6**).
